# Detection and Isolation of Psychrotrophic *Acinetobacter* spp. in Raw Milk from Ecuador: A Hidden Microbial Risk for Dairy Products

**DOI:** 10.3390/foods15152662

**Published:** 2026-07-29

**Authors:** Andrea Padilla-Cerda, Anthony Loor-Giler, Byron Puga-Torres, Silvana Santander-Parra, Luis Núñez

**Affiliations:** 1 Facultad de Ingeniería y Ciencias Aplicadas, Carrera de Ingeniería en Biotecnología, Universidad de Las Américas (UDLA), Antigua Vía a Nayon S/N, Quito EC 170124, Ecuador; andrea.padilla.cerda@outlook.com; 2 Laboratorios de Investigación, Dirección General de Investigación, Universidad de las Américas (UDLA), Antigua Vía a Nayón S/N, Quito EC 170124, Ecuador; a.abel.loor.giler@gmail.com; 3 Facultad de Ciencias Veterinarias, Universidad de Buenos Aires, Av. Chorroarín 280, Buenos Aires 1427, Argentina; 4 Facultad de Medicina Veterinaria y Zootecnia, Universidad Central del Ecuador, Jerónimo Leyton s/n y Gilberto Gatto Sobral, Quito EC 170521, Ecuador; bpuga@uce.edu.ec; 5 Facultad de Ciencias de la Salud, Carrera de Medicina Veterinaria, Universidad de Las Américas (UDLA), Antigua Vía a Nayon S/N, Quito EC 170124, Ecuador; silvanahsp@yahoo.com; 6 One Health Research Group, Facultad de Ciencias de la Salud, Universidad de Las Américas (UDLA), Quito EC 170124, Ecuador

**Keywords:** *Acinetobacter* spp., raw milk, psychrotrophic bacteria, milk spoilage

## Abstract

*Acinetobacter* species are psychrotrophic microorganisms commonly associated with the spoilage of refrigerated raw milk and dairy products; the aim of this study is to determine the prevalence and spreading of *Acinetobacter* species isolated from raw milk collected in Ecuador. During the dry and rainy seasons, 400 raw milk samples were collected from dairy farms in the provinces of Pichincha and Manabí. Following genus-level qPCR detection, species-specific PCR screening detected to ten targeted *Acinetobacter* species in the enriched raw milk samples. Microbiological isolation was followed by *16S rRNA* gene sequencing to confirm that the recovered colonies belonged to the genus *Acinetobacter* and to obtain preliminary taxonomic assignments. Correlations between bacterial presence and climatic season, province and farm size were analyzed; *Acinetobacter* spp. was detected in 342 of the 400 enriched raw milk samples (85.5%). A higher prevalence was observed in samples from Pichincha province, with *A. lwoffii* being the most prevalent species (88.89% of positive samples). Five species were isolated: *A. lwoffii*, *A. baumannii*, *A. calcoaceticus*, *A. pittii* and *A. haemolyticus*. The high prevalence and diversity of *Acinetobacter* species in Ecuadorian raw milk suggest widespread distribution of this genus in local dairy systems, highlighting its potential role in milk spoilage and reduced quality.

## 1. Introduction

Milk is a nutrient-dense food imperative for human nutrition and it sustains dairy production systems; however, these characteristics make it highly perishable [1]. The dairy sector is a major economic activity in developing countries, driven by high consumption and varied production scales [2]. In Ecuador, the dairy industry generates about $1.4 billion annually and produces 5.5 million liters of raw milk daily; majority of this production is concentrated in Pichincha and Manabí [3,4,5,6]. The Survey of Agricultural Land Area and Production (ESPAC) reports that Pichincha is the largest milk-producing province in the Andean region and Manabí the largest producer in the coastal region; likewise, 77% of cow’s milk is sold in liquid form [7]. Of this, nearly 50% is distributed through informal channels that lack sanitary supervision, where most of this milk is considered unfit for human consumption, as it largely fails to comply with the provisions of the Ecuadorian Technical Standard (NTE) INEN 9 [8,9].

Raw milk, defined by its lack of pasteurization, is particularly susceptible to microbial contamination and spoilage, because its nutrient composition and neutral pH support the growth of diverse microorganisms [10,11]. Over 160 bacterial species have been identified in raw milk, where environmental conditions, hygiene, and milking techniques affect bacterial loads [12,13,14]. Refrigeration at 4 to 10 °C limits mesophilic and thermophilic growth, but psychrotrophic species can still proliferate under cold storage conditions [15,16,17]. The predominant bacteria in refrigerated milk include *Pseudomonas*, *Acinetobacter*, *Aeromonas*, *Bacillus*, and *Mycobacterium* [15]. Despite the economic relevance of the dairy sector in Ecuador, studies addressing the diversity of spoilage-associated bacteria such as *Acinetobacter* spp. remains limited [18].

The genus *Acinetobacter* currently includes approximately 90 validly published species, as recognized by the List of Prokaryotic names with Standing in Nomenclature (LPSN). These bacteria are Gram-negative, aerobic, and non-fermentative, distributed in the environment and on animal skin, including bovine udders [19,20,21,22]. Some *Acinetobacter* species produce thermostable enzymes, such as proteases and lipases, which cause proteolysis and lipolysis, generally undesirable in milk and dairy products [23]. Proteolytic activity is especially important because casein accounts for about 80% of milk’s total protein. Its breakdown reduces nutritional quality, sensory properties, and milk’s processing capabilities [24].

According to the Pasteurized Milk Ordinance, it is required to contain a maximum of 100,000 CFU/mL; however, studies have shown that proteolysis can occur even at low bacterial counts, resulting in a loss of up to 20% of κ-casein after two days at 5 °C [17,25]. *Acinetobacter* spp. have been detected among psychrotrophic bacteria isolated from raw milk in several countries, including Spain, South Korea, China, and Brazil [16,26,27,28]. However, their occurrence and diversity in Ecuadorian raw milk have not been characterized.

*Acinetobacter* spp. in raw milk represent a central risk to quality and dairy processing in Ecuador. Whilst monitoring has been limited and research has focused primarily on pathogens, this study addresses the crucial knowledge gap by estimating the molecular prevalence of *Acinetobacter* spp. in Ecuadorian raw milk. This is of particular importance for the effective prevention of spoilage bacteria and for the improvement of milk safety and quality controls within Ecuador’s dairy sector.

## 2. Materials and Methods

### 2.1. Sampling

Between September 2022 and July 2023, 400 raw milk samples (100 mL) were obtained from cooling tanks at cattle farms and containers used for the direct sale of raw milk in local markets. Sampling was conducted in Pichincha and Manabí in collaboration with the School of Veterinary Medicine and Animal Science of Universidad Central del Ecuador. (UCE). The 100 mL volume corresponded to the collected laboratory sample and provided sufficient material for homogenization, subsampling, and the molecular and culture-based procedures included in the study. Samples were stored in sterile recipients, maintained at 4 °C during transportation, and processed at the research laboratories of Universidad de Las Américas (UDLA). Sample collection and handling followed the general recommendations of NTE INEN-ISO 707 and ISO 7218:2024 [29,30].

The minimum sample size required to estimate the prevalence of *Acinetobacter* spp. in raw milk from Ecuador was calculated using the following formula:

(1) $$n=\frac{Z^{2}\times P_{exp}\times \left(1-P_{exp}\right)}{d^{2}}$$

An expected prevalence of *Acinetobacter* spp. of 50% was assumed. The sample size (n) was determined using an assumed prevalence (*Pexp*) of 0.50, a precision level (*d*) of 0.05, and the *Z* value corresponding to a 95% confidence level [31]. Based on this calculation, 200 samples were collected from Pichincha and 200 from Manabí. The sampling population included small (*n* = 147), medium (*n* = 137), and large (*n* = 116) dairy producers. Producers were classified according to herd size as small (1–20 dairy cows), medium (21–100 dairy cows), or large (>100 dairy cows) [32]. Climatic periods were assigned using information from the National Institute of Meteorology and Hydrology. Samples collected from June to August were classified within the dry season (*n* = 269), whereas those collected from September to May were classified within the rainy season (*n* = 131) (Supplementary Materials—Table S1).

All procedures were conducted under institutional regulations and the authorization issued by the Ecuadorian Agency for Phytosanitary and Zoosanitary Regulation and Control (AGROCALIDAD; approval INT/DA/019, 31 January 2018).

### 2.2. Cold Enrichment and DNA Extraction

Raw milk samples were subjected to a cold enrichment, and 4 mL of raw milk was combined with 36 mL of Brain Heart Infusion (BHI) broth (Difco™, Fisher Scientific, Waltham, MA, CA 237500, USA). The resulting 40 mL enrichment, containing 10% (*v*/*v*) raw milk, was maintained at 8 °C for 72 h under orbital agitation at 150 rpm.

After incubation, bacterial cells were collected by centrifugation at 4000× *g* for 15 min. The supernatant was discarded, and the pellet was suspended in 200 µL of Tris-EDTA buffer. Cell disruption was promoted by freezing the suspension at −80 °C for 10 min, followed by heating at 56 °C for 1 min. Subsequently, 600 µL of guanidinium thiocyanate solution was added, and the mixture was gently inverted and maintained at room temperature for 5 min.

For the separation phase, 100 µL of chloroform was added, followed by gentle mixing and centrifugation at 12,000× *g* for 5 min at 4 °C. The upper aqueous phase was transferred to a new tube containing 500 µL of isopropanol and maintained overnight at −20 °C to precipitate the nucleic acids. The resulting DNA pellet was washed three times with 70% ethanol, air-dried, and dissolved in 30 µL of TE buffer by incubation at 56 °C for 15 min. DNA extraction was performed following a previously described phenol–chloroform protocol [33].

### 2.3. Genus-Level qPCR Detection of Acinetobacter spp.

Genus-level detection was performed in a CFX96 Touch Real-Time PCR Detection System (Bio-Rad Laboratories, Hercules, CA, USA) using a SYBR Green assay. The 10 µL reaction mixture comprised PowerUp™ SYBR™ Green Master Mix (Thermo Fisher Scientific, Carlsbad, CA, USA), 0.4 µM of each genus-specific primer (Table 1), 2 µL of DNA extracted from the enriched milk sample, and nuclease-free water. Each sample was analyzed in duplicate. DNA from *A. baumannii* ATCC 19606 and nuclease-free water were included as the positive and no-template controls, respectively.

Thermocycling conditions consisted of UDG activation at 50 °C for 2 min and enzyme activation at 95 °C for 2 min, followed by 40 cycles at 95 °C for 30 s and 58 °C for 30 s. A melting-curve analysis was performed immediately after amplification. Samples were classified as positive when both replicates showed exponential amplification and a single melting peak within the experimentally established range of 55.5–56.5 °C. Reactions were conducted using a CFX96 Touch Real-Time PCR Detection System (Bio-Rad Laboratories, Hercules, CA, USA).

### 2.4. Species-Specific Molecular Screening of Enriched Milk Samples by End-Point PCR

Genus-positive enriched milk samples were further screened by end-point PCR for species specificity, using previously published primers that target ten *Acinetobacter* species most associated with food contamination or spoilage (Table 1). This molecular screening was performed before bacterial isolation to determine the species potentially present in each enriched sample and to prioritize samples for subsequent culture-based recovery.

Each PCR assay was performed in a final volume of 10 µL containing GoTaq^®^ Green Master Mix 2X (Promega Corporation, Madison, WI, USA), that is, species-specific primers at a final concentration of 0.3 µM, nuclease-free water, and 2 µL of DNA template or nuclease-free water for no-template control. PCR amplification parameters were established according to the conditions described by the original authors for each primer pair [35,36]. The protocol included an initial denaturation phase at 95 °C for 30 s followed by 40 amplification cycles consisting of denaturation at 94 °C for 30 s, annealing at the corresponding primer-specific temperature, and extension at 72 °C for 30 s. The PCR products were separated by horizontal electrophoresis on 2% (*w*/*v*) agarose gels. The gels were then stained with SYBR Safe DNA Gel Stain (Thermo Fisher Scientific, Carlsbad, CA, USA) and visualized using a UV transilluminator (Bio-Rad Laboratories, Inc., Hercules, CA, USA). The size of the amplicons was determined by comparison with a DNA ladder (50 bp or 100 bp) (Invitrogen, Thermo Fisher Scientific, Carlsbad, 170 California, USA). Each genus-positive enriched milk sample was tested independently with the ten species-specific PCR assays. Therefore, a single sample could yield positive results for more than one targeted *Acinetobacter* species. The species-specific PCR profiles were subsequently used to prioritize enriched samples for bacterial isolation.

### 2.5. Bacteria Isolation and DNA Extraction

Enriched milk samples were selected for culture-based isolation by prioritizing those positive for a single *Acinetobacter* target. When single-target samples were unavailable, samples with the fewest additional species-specific detections were selected (Supplementary Materials—Table S3).

Selected enriched samples were cultured on MacConkey Agar (Difco™, Fisher Scientific, Massachusetts, CA 212123, USA) supplemented with cefotaxime (2 mg/L) and vancomycin (10 mg/L), a selective medium used to favor the recovery of *Acinetobacter* spp. while limiting the growth of competing Gram-negative and Gram-positive microorganisms [37,38]. After incubation, colonies showing morphology compatible with *Acinetobacter* spp., including smooth, circular, translucent to pale, and non-lactose-fermenting colonies, were aseptically selected and suspended in 200 µL of TE buffer in sterile 1.5 mL microcentrifuge tubes for DNA extraction following the boiling method [39]. Bacterial suspensions were homogenized by vortexing and maintained at −20 °C overnight to facilitate cell disruption. Samples were subsequently exposed to thermal lysis at 95 °C for 1 min using a dry bath, vortexed again, and centrifuged at 10,000× *g* for 10 min at 4 °C. Finally, 100 µL of the resulting supernatant was recovered and transferred to 0.2 mL PCR tubes.

### 2.6. 16S rRNA Gene Sequencing and Taxonomic Characterization of Recovered Isolates

Genomic DNA obtained from isolated bacterial colonies was used for amplification of the *16S ribosomal RNA* gene. A fragment of approximately 1400 bp was amplified by PCR using the universal primers 27F and 1492R [40]. PCR reactions were performed in a final volume of 25 µL containing GoTaq^®^ Green Master Mix 2X (Promega Corporation, Madison, WI, USA), 0.3 µM of each primer, 2 µL of DNA template, and nuclease-free water. Amplification was carried out according to the manufacturer’s recommendations using 40 amplification cycles and an annealing temperature of 55 °C.

Amplicons were analyzed by agarose gel electrophoresis and visualized using SYBR™ Safe DNA Gel Stain (Invitrogen™, Thermo Fisher Scientific, Carlsbad, CA, USA). PCR products were subsequently purified using the ExoSAP-IT™ PCR Product Cleanup Reagent (Applied Biosystems, Carlsbad, CA, USA). Purified products were subjected to sequencing using the BigDye^®^ Terminator v3.1 Cycle Sequencing Kit (Applied Biosystems, Santa Clara, CA, USA), followed by capillary electrophoresis in an ABI 3500 Genetic Analyzer (Applied Biosystems, Foster City, CA, USA). The obtained electropherograms were edited and analyzed using Geneious Prime^®^ software version 10.2.3 (https://www.geneious.com) (Biomatters Ltd., Auckland, New Zealand; Geneious Prime). Sequence identity was confirmed through comparison with reference sequences available in the GenBank database using BLASTn version 2.13.0. The partial *16S rRNA* gene sequences generated in this study were deposited in the GenBank database under accession numbers PZ695520–PZ695536 (Table 2).

Multiple sequence alignment was performed using the MUSCLE v3.8 implemented in MEGA X [41]. Phylogenetic relationships were inferred using the Neighbor-Joining statistical method, while genetic distances were calculated using the p-distance approach and branch support was evaluated by a bootstrap phylogeny test with 1000 replicates. This species-level identification is based on the principle of sequence similarity and the utilization of phylogenetic clustering. It is important to acknowledge that the discriminatory power of partial *16SrRNA* gene sequences is limited for closely related *Acinetobacter* species. Gene sequencing was used to verify that the recovered colonies belonged to the genus *Acinetobacter* and to provide a preliminary taxonomic assignment.

### 2.7. Statistical Analysis

Descriptive statistics were used to summarize the frequency and percentage of *Acinetobacter*-positive samples according to province, climatic conditions, producer size, and identified species. Associations between the presence of *Acinetobacter* spp. and the categorical variables evaluated (province, climate, and producer size) were analyzed using the Chi-square test (χ^2^). Odds ratios (OR) with 95% confidence intervals (95% CI) were additionally calculated to estimate the strength of association between the evaluated variables and *Acinetobacter* occurrence. All statistical tests were carried out using RStudio software version 4.4.3 (https://www.R-project.org/) (R Core Team, Vienna, Austria).

## 3. Results

### 3.1. Molecular Detection of Acinetobacter spp. in Enriched Raw Milk Samples by qPCR

Of the 400 enriched raw milk samples analyzed, 342 were positive for *Acinetobacter* spp. by qPCR, corresponding to an overall molecular detection rate of 85.5% (342/400). In Pichincha, 94% (188/200) of the samples tested positive for the genus, while in Manabí the presence of the bacteria was found in 77% (154/200) of raw milk samples (Table 2); statistically significant differences were found between the province and the presence of *Acinetobacter* spp. (*p* < 0.05), with the province of Pichincha showing a higher probability for positive results in comparison to those from Manabí (OR = 4.62; 95% CI: 2.39–9.15). In contrast, the proportion of positive samples varied according to producer size, with small farms having 86.4% (127/147) positive samples, medium farms having 85.4% (117/137) positive samples, and large farms having 84.5% (98/116) positive samples, with no significant association between producer size and the presence of these bacteria (*p* = 0.908).

**Figure 1:**
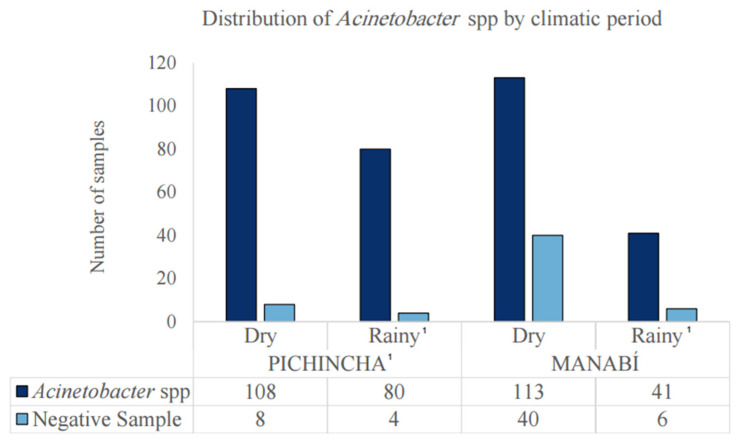
Detection of *Acinetobacter* spp. in raw milk by province and climate period. ^1^ Statistically significant difference.

During the dry season, 82.16% (221/269) of enriched raw milk samples tested positive for *Acinetobacter* spp., compared to 92.37% (121/131) during the rainy season. The Chi-square test showed a significant association between climatic period and the detection of *Acinetobacter* spp. (*p* < 0.05). Odds ratio analysis indicated differences in the likelihood of *Acinetobacter* spp. detection between dry and rainy conditions (OR = 0.39; 95% CI: 0.19–0.78) (Figure 1).

### 3.2. Species-Specific Molecular Detection in Genus-Positive Enriched Milk Samples

The distribution of *Acinetobacter* species in 342 genus-positive enriched raw milk samples exhibited consistent variation according to province and producer size (Table 3). Individual samples could test positive for more than one targeted *Acinetobacter* species (Supplementary Materials—Table S1). *A. lwoffii* was the most prevalent species, with 85.8% (304/342) positive samples, representing the highest proportion in both provinces. This was followed by *A. johnsonii*, *A. baumannii*, *A. ursingii*, and *A. pittii*, which also exhibited relatively high frequencies. It is evident that less common species, including *A. nosocomialis*, *A. haemolyticus*, *A. calcoaceticus*, *A. bereziniae*, and *A. schindleri*, displayed low detection rates and did not show notable distribution patterns (Table 4). The statistical analysis indicated that *A. baumannii* exhibited a significantly higher likelihood of detection in Pichincha compared with Manabí. In contrast, species such as *A. lwoffii*, *A. ursingii*, *A. nosocomialis*, and *A. schindleri* were more likely to be identified in Manabí. The remaining species demonstrated no significant correlation between their detection and the province of origin (*p* > 0.05).

The distribution of *Acinetobacter* according to producer size exhibited consistent patterns. *A. lwoffii* was the most prevalent species in all three strata, with 85.8% (109/127) positives in small producers, 85.5% (100/117) in medium producers, and 96.9% (95/98) in large producers. Other species that exhibited a notable prevalence included *A. johnsonii*, *A. ursingii*, and *A. baumannii*, which in numerous instances attained proportions approaching 20–25%, indicative of their consistent circulation across the various production strata. In contrast, species such as *A. nosocomialis*, *A. pittii*, *A. calcoaceticus*, *A. bereziniae*, and *A. schindleri* were detected less frequently and constituted the least represented group among producers. Statistical analysis revealed a significant association with producer size (*p* < 0.05); *A. lwoffii* (OR = 0.19; 95% CI: 0.05–0.67) and *A. bereziniae* (OR = 0.26; 95% CI: 0.08–0.83) were less frequently detected in small and medium producers than in large producers.

**Figure 2:**
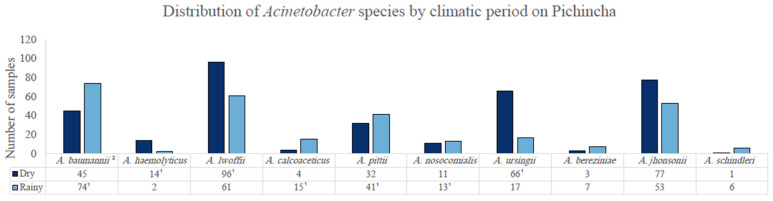
Species-specific PCR detection of *Acinetobacter* spp. in Pichincha during the dry (*n* = 108) and rainy (*n* = 80) seasons. Bars represent the absolute number of PCR-positive enriched raw milk samples. Individual samples could be positive for more than one species-specific target. ^1^ Statistically significant difference according to climatic period. ^2^ Statistically significant difference according to province.

The distribution of *Acinetobacter* species in Pichincha varied between the dry and rainy seasons (Figure 2). *A. lwoffii* was the most frequently detected species, occurring in 88.9% (96/108) of genus-positive samples collected during the dry season and 76.3% (61/80) during the rainy season. Other frequently detected species included *A. ursingii*, which was found in 61.1% (66/108) and 21.3% (17/80) of samples during the dry and rainy seasons, respectively, and *A. johnsonii*, detected in 71.3% (77/108) and 66.3% (53/80), respectively. In contrast, *A. calcoaceticus*, *A. bereziniae*, and *A. schindleri* were detected at lower frequencies and showed no substantial variation between climatic periods.

**Figure 3:**
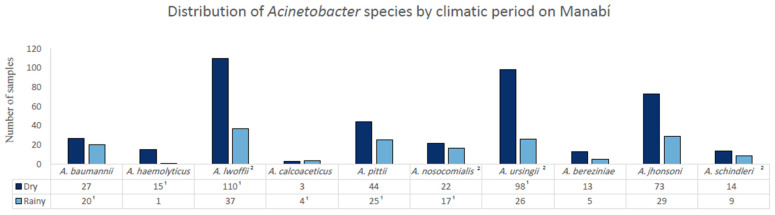
Species-specific PCR detection of *Acinetobacter* spp. in Manabí during the dry (*n* = 113) and rainy (*n* = 41) seasons. Bars represent the absolute number of PCR-positive enriched raw milk samples. Individual samples could be positive for more than one species-specific target. ^1^ Statistically significant difference according to climatic period. ^2^ Statistically significant difference according to province.

In Manabí, the climatic conditions were identified as a significant factor influencing species distribution (Figure 3). The analysis revealed that dry weather conditions accounted for approximately 57% of all *A. lwoffii* detections and almost 60% of *A. pittii* positives within the province. In contrast, the remaining species, including *A. baumannii*, *A. haemolyticus*, *A. calcoaceticus*, *A. bereziniae*, *A. johnsonii* and *A. schindleri*, did not demonstrate a significant association with climate in this province. It is evident that the observed patterns in Manabí demonstrate a discernible climatic influence on certain species, while others exhibit a more equitable distribution across both dry and rainy periods.

Statistical analysis demonstrated a significant association between the climatic period and the presence of the majority of species, with the exception of *A. bereziniae, A. johnsonii*, and *A. schindleri. A. lwoffii*, *A. haemolyticus*, and *A. ursingii* exhibited a higher propensity for detection during the dry season, while the remaining species demonstrated a reduced likelihood of occurrence during this period.

**Table 4:** Distribution of *Acinetobacter* species detected by end-point PCR in positive raw milk samples for *Acinetobacter* spp. by province and producer size; percentages were calculated within each province–producer size group. Percentages within each province–producer size row were calculated using the number of genus-positive samples in the corresponding subgroup as the denominator. Percentages in the total row were calculated using all 342 genus-positive samples.

Province	Producer Size	*A. baumannii*	*A. haemolyticus*	*A. lwoffii* ^1^	*A. calcoaceticus*	*A. pittii*	*A. nosocomialis* ^1^	*A. ursingii* ^1^	*A. bereziniae*	*A. jhonsonii*	*A. schindleri* ^1^	Total Samples
**PICHINCHA**	**Small**	41 (56.16%) ^1^	10 (13.70%)	58 (79.45%)	5 (6.85%)	27 (39.99%)	11 (15.07%)	38 (52.05%)	0 (0.00%)	49 (67.12%)	1 (1.37%)	73
	**Medium**	48 (70.59%) ^1^	4 (5.88%)	53 (77.94%)	6 (8.82%)	30 (44.12%)	6 (8.82%)	23 (33.82%)	5 (7.35%)	45 (66.18%)	2 (2.94%)	68
	**Large**	30 (63.83%) ^1^	2 (4.26%)	46 (97.87%) ^1^	8 (17.02%)	16 (34.04%)	7 (14.89%)	22 (46.81%)	5 (10.64%) ^1^	36 (76.60%)	4 (8.51%)	47
**MANABÍ** ^1^	**Small**	19 (35.19%)	6 (11.11%)	51 (94.44%)	2 (3.70%)	21 (38.89%)	13 (24.07%)	46 (85.19%)	4 (7.41%)	35 (64.81%)	8 (14.81%)	54
	**Medium**	12 (24.49%)	5 (10.20%)	47 (95.92%)	2 (4.08%)	22 (44.90%)	13 (26.53%)	38 (77.55%)	8 (16.33%)	31 (63.27%)	10 (20.41%)	49
	**Large**	16 (31.37%)	5 (9.80%)	49 (96.08%) ^1^	3 (5.88%)	26 (50.98%)	13 (25.49%)	40 (78.43%)	6 (11.76%) ^1^	36 (70.59%)	5 (9.80%)	51
**Total Positives**		166 (48.54%)	32 (9.36%)	304 (88.89%)	26 (7.60%)	142 (41.52%)	63 (18.42%)	207 (60.53%)	28 (8.19%)	232 (67.84%)	30 (8.77%)	342

^1^ Statistically significant difference.

### 3.3. Isolation and 16S Sequencing of Acinetobacter Species

A total of 18 presumptive *Acinetobacter* isolates were recovered. *A. baumannii* (*n* = 9), *A. lwoffii* (*n* = 6), *A. calcoaceticus* (*n* = 1), *A. haemolyticus* (*n* = 1), and *A. pittii* (*n* = 1). No isolates corresponding to *A. nosocomialis*, *A. ursingii*, *A. johnsonii*, *A. schindleri*, and *A. bereziniae* were detected. The partial sequencing of the *16S rRNA* gene indicated that the isolates obtained in this study exhibited NT similarities of over 98.5% (Supplementary Materials—Table S2) compared to reference sequences of the same species, which were deposited in the GenBank database.

**Figure 4:**
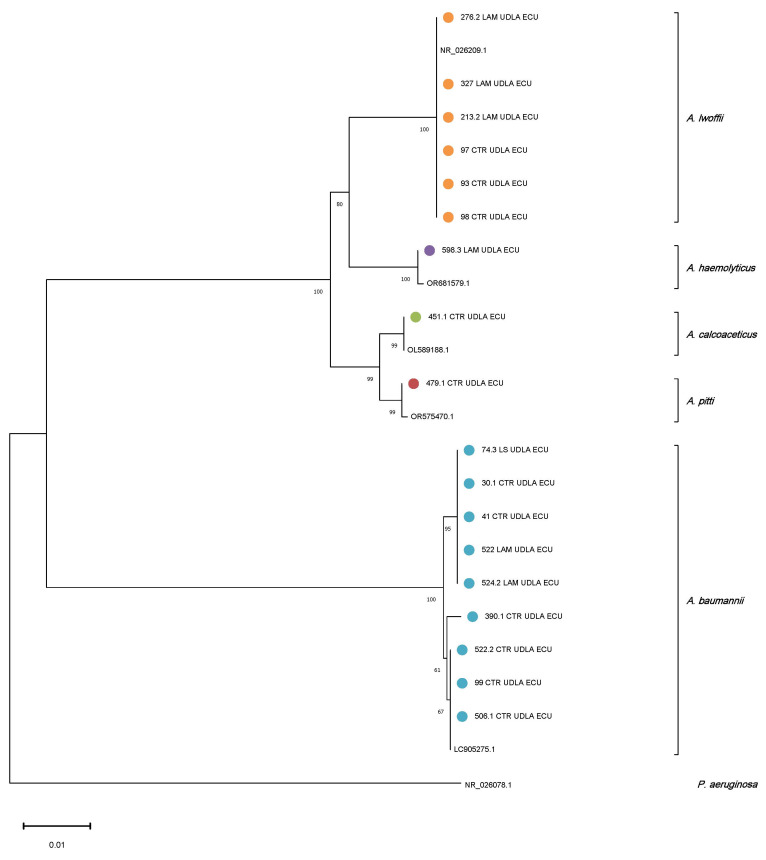
Phylogenetic tree among *Acinetobacter* isolates obtained in the present study and reference strains retrieved from NCBI was inferred using partial *16S rRNA* gene sequences. Sequence alignment was carried out with MUSCLE v3.8, followed by phylogenetic reconstruction using the Neighbor-Joining approach. Branch support was evaluated by bootstrap analysis with 1000 replicates, and bootstrap values are displayed at the corresponding nodes. The scale bar indicates the number of substitutions per site. *Pseudomonas aeruginosa 16 rRNA* gene sequence was included as an outgroup. Isolates generated in this study are marked with dots (●).

The phylogenetic tree (Figure 4) illustrated a clear segregation of the isolates recovered into specific clades for each species within the genus *Acinetobacter*. The isolates assigned to *A. baumannii* and *A. lwoffii* were consistently grouped with their respective reference sequences, while the isolates corresponding to *A. calcoaceticus*, *A. haemolyticus*, and *A. pittii* were grouped into distinct clades associated with each species.

## 4. Discussion

*Acinetobacter* spp. have become increasingly important in recent decades due to their antimicrobial resistance and the pathogenic role of certain species, such as *A. baumannii* [19]. However, its consistent presence in animal matrices, such as milk, indicates its notable environmental ubiquity [42]. This bacterial genus is widely distributed in soil, water, industrial equipment, and humid environments, and can also form part of the epithelial, respiratory, and digestive microbiota of farm animals [43]. Several studies have documented the presence of *Acinetobacter* spp. in raw milk: for instance, 7.7% of bulk milk samples were found to be contaminated [28]. Furthermore, relative abundances ranging from 4.9% to 20.3% have been reported, depending on geographical location, season, and milk type [44,45,46].

Consequently, the high prevalence of *Acinetobacter* spp. observed in this study (85.5%) exceeds the detection frequencies reported in most previous studies and suggests substantial dissemination of this genus within the Ecuadorian production system. Comparable studies have identified *Acinetobacter* as a dominant genus in the microbiota of raw milk, especially under refrigerated storage conditions, where its relative abundance can increase significantly over time [44,47]. This high prevalence may be associated with *Acinetobacter*’s ability to persist in cold environments, such as those created by milk refrigeration, and colonize milking and storage surfaces [47,48]. Taken together, these findings support the hypothesis that *Acinetobacter* spp. may constitute a dominant and stable component of the raw milk microbiota, thereby reinforcing their relevance for evaluating milk quality and for assessing their potential as contributors to spoilage-related industrial processes.

The observed variations in the prevalence of *Acinetobacter* spp. among different sampling sites may be related to differences in dairy production systems, including milking practices, storage conditions, and hygiene management [49]. In the present study, *Acinetobacter* spp. showed a higher prevalence in samples from Pichincha than from Manabí, which may be associated not only with the intensive dairy activity of Pichincha, Ecuador’s main milk-producing region, but also with its colder environmental conditions, which may favor the survival and proliferation of psychrotrophic bacteria such as *Acinetobacter* spp. [7,32]. Previous studies have reported that the presence and persistence of environmental bacteria, including *Acinetobacter*, in raw milk are influenced by factors such as refrigeration infrastructure, water quality, and sanitation procedures along the milk production chain [14]. Additionally, the environmental ubiquity of this genus in soil, water, and farm-associated surfaces contributes to its widespread distribution across dairy systems, regardless of production scale [21]. Consistently, the lack of a clear association between producer size and *Acinetobacter* prevalence observed in this study supports the notion that this genus is broadly distributed across dairy farms of different production scales.

Conversely, climatic conditions showed a measurable effect on the distribution of *Acinetobacter* spp., as reflected by differences between the dry and rainy seasons. Similar seasonal patterns have been reported in studies of raw milk microbiota, where *Acinetobacter* was consistently detected as part of the core microbiota and showed higher relative abundances during warmer periods [44,50,51]. This behavior is strongly associated with the psychrotrophic nature of the genus, which enables survival and growth under refrigeration through physiological adaptations, such as modifications in membrane lipid composition, that maintain membrane fluidity at low temperatures [46,47,48]. In contrast, producer size did not significantly influence the occurrence of *Acinetobacter* spp., a finding that aligns with studies reporting comparable psychrotrophic bacterial loads across farms with different production scales [51]. This lack of association may be explained by the ubiquitous environmental distribution of *Acinetobacter* in water, soil, teat surfaces, and milking equipment, which promotes recurrent contamination regardless of herd size or production volume [28,42,48]. Together, these observations suggest that seasonal environmental conditions and routine management practices exert a stronger influence on the presence and persistence of *Acinetobacter* spp. in raw milk than producer size alone.

Among the detected species, *A. lwoffii* was the most prevalent, a pattern consistent with reports indicating that the former *A. lwoffii* biotype dominates microbial communities in food products [52]. *A. lwoffii* adaptation to diverse ecological niches, including soil, water, milking environments, and the gastrointestinal and respiratory microbiota, combined with its tolerance to nutrient limitation, desiccation, and temperature fluctuations, supports its recurrent detection in raw milk [21]. Recent studies have reported an increasing detection rate of *A. lwoffii* in bovine milk, particularly associated with subclinical mastitis and biofilm formation in dairy environments, highlighting its persistence within the mammary ecosystem [52,53]. Other frequently detected species included *A. johnsonii*, *A. ursingii*, and *A. baumannii*, all of which exhibit cold-tolerance mechanisms that facilitate persistence during storage and refrigeration, a characteristic commonly reported for psychrotrophic *Acinetobacter* species isolated from raw milk [54,55]. *A. johnsonii* has been repeatedly associated with refrigerated aquatic foods and behaves as a specific spoilage organism, with its cold adaptation enhanced through interactions with other bacteria such as *Shewanella putrefaciens*, suggesting environmental suitability under low-temperature food processing [56]. The higher likelihood of detecting *A. baumannii* in Pichincha may reflect contamination routes associated with milking practices and equipment hygiene, as this species has been widely associated with contamination of teats, udder surfaces, and milking systems in bulk tank milk [28]. In contrast, the greater presence of *A. johnsonii* and *A. ursingii* in Manabí aligns with their frequent isolation from water systems and humid environments, supporting dissemination through water-associated food chains in coastal regions [42,57].

The distribution of individual species exhibited heterogeneous patterns, primarily influenced by producer scale and management conditions. *A. lwoffii* demonstrated a persistent distribution across diverse production systems, yet it exhibited a heightened prevalence in large farms, where milking and storage processes rely heavily on automated machinery and centralized cooling systems [58]. This pattern is consistent with the strong biofilm-forming capacity previously reported for *A. lwoffii* on abiotic surfaces, including stainless-steel equipment, where it can persist despite exposure to phenolic sanitizers and commonly used disinfectants [53], a trait that has also been documented for food-derived *Acinetobacter* isolates showing tolerance to industrial sanitizing agents [57]. Conversely, species such as *A. bereziniae* exhibited a higher propensity to manifest in industrial environments, implying that shared equipment, tank geometry, and standardized cooling systems may favor environmental persistence [59], as previously observed for *Acinetobacter* spp. in bulk tank milk and processing lines [28,50]. These observations corroborate earlier evidence indicating that *Acinetobacter* species can persist in milking devices, pipelines, and storage tanks, emphasizing that hygiene practices, equipment sanitation, and environmental control represent more decisive drivers of species-level occurrence in raw milk than farm size alone [42,48].

Culture-dependent recovery revealed a marked reduction in species diversity compared to molecular detection performed on DNA extracted from cold-enriched milk, underscoring the selective nature of microbiological isolation in complex matrices such as raw milk. Although PCR-based analysis indicated the presence of all ten targeted *Acinetobacter* species, only five were successfully isolated and confirmed by sequencing, with *A. baumannii* and *A. lwoffii* predominating among the recovered isolates. This outcome may be partially influenced by the use of MacConkey agar during the isolation process, as this medium is conventionally employed for the recovery of *A. baumannii* and has been shown to favor its growth due to its high adaptability to conventional selective media [37,38]. This *Acinetobacter* species is frequently recovered using MacConkey agar in both clinical and environmental surveillance studies, often outperforming other *Acinetobacter* species in culturability [37,38]. This pattern is consistent with previous studies reporting that these species display higher culturability and survival under dairy-associated stressors, including low temperatures, nutrient limitation, and prolonged storage [44,55]. Particularly, *A. baumannii* has been repeatedly shown to grow efficiently under refrigeration and tolerate adverse environmental conditions, increasing its likelihood of recovery during conventional isolation procedures [54]. Similarly, *A. lwoffii* is frequently described as one of the most persistent and readily culturable members of the genus in milk and processing environments, a feature attributed to its broad metabolic capacity and enhanced environmental suitability on abiotic surfaces commonly associated with milking and storage systems [48].

In contrast, other *Acinetobacter* species detected molecularly may remain at low population levels, exhibit slower growth kinetics, or enter physiological states that limit their recovery on standard culture media, as previously observed in dairy ecosystems [49]. Some cells may also have entered a viable but non-culturable state under refrigeration, nutrient limitation, or other environmental stresses, allowing their DNA to be detected while preventing colony formation under the culture conditions used [60]. Conversely, PCR-based detection does not demonstrate cellular viability and may amplify DNA remaining from dead or membrane-damaged bacterial cells [61]. To enhance the recovery of a broader diversity of *Acinetobacter* species, future studies could incorporate alternative or complementary culture media, such as chromogenic media specifically designed for *Acinetobacter* detection, including CHROMagar™ *Acinetobacter* or Leeds *Acinetobacter* Medium (LAM), which have demonstrated improved sensitivity and species coverage compared to MacConkey agar alone [37,38]. Phylogenetic analysis based on partial *16S rRNA* gene sequencing supported the assignment of the recovered colonies to the genus *Acinetobacter*, this marker does not provide sufficient resolution for definitive identification of several closely related species within the genus [62]. Therefore, the species-level assignments reported for the isolates should be considered presumptive. The absence of sequencing of higher-resolution markers, such as *rpoB* or *gyrB*, represents a limitation of the present study [62,63].

This study has several limitations that should be considered when interpreting the findings. Although the sample size was calculated to estimate *Acinetobacter* molecular prevalence with a 95% confidence level and 5% precision, sampling was conducted in Pichincha and Manabí, two major dairy-producing provinces representing the Andean and coastal regions of Ecuador. Therefore, extrapolation of the findings to dairy systems in other Ecuadorian regions should be made cautiously. In addition, the number of samples collected differed between climatic periods, which may influence comparisons based on absolute frequencies. The cold-enrichment procedure improved the detection of *Acinetobacter* DNA but did not allow quantification of the bacterial concentration originally present in raw milk and may have differentially favored taxa according to their growth characteristics. Culture-based recovery relied on a selective medium and was not designed to provide an exhaustive representation or species-specific recovery rate for all molecularly detected taxa. Finally, taxonomic characterization of the recovered isolates was based on partial *16S rRNA* gene sequencing, which has limited discriminatory resolution among closely related *Acinetobacter* species. Future studies should include additional dairy-producing regions, more balanced longitudinal sampling, quantitative bacterial enumeration, complementary culture conditions, and higher-resolution taxonomic markers.

## 5. Conclusions

This study demonstrates a high prevalence of *Acinetobacter* spp. in raw milk from Pichincha and Manabí, with marked differences associated with geographic location and seasonal conditions. Molecular detection revealed a broader species diversity than culture-based approaches, highlighting the widespread presence of this genus and the selective nature of microbiological isolation. The predominance of *A. lwoffii* and *A. baumannii* reflects their environmental persistence and enhanced suitability for survival under dairy-associated conditions. These findings emphasize the importance of improved hygiene practices and molecular surveillance to better assess the ecological and technological impact of *Acinetobacter* spp. in raw milk.

## Figures and Tables

**Table 1:** Primers used for molecular detection of *Acinetobacter* spp. and identification of 10 different species of the genus.

Bacteria	Primer Name	Sequence (5′-3′)	Amplicon Length (Pb)	Reference
*Acinetobacter* spp. ^1^	Ac696F	TAYCGYAAAGAYTTGAAAGAAG	107	[34]
	Ac1093R	CMACACCYTTGTTMCCRTGA		
*A. calcoaceticus*	D14	GACAACAGTTATAAGGTTTCAGGTG	428	[35]
	D19	CCGCTATCTGTATCCGCAGTA		
*A. pittii*	Apit-F	TGGGCAGTTACCAGATTGACCTA	147	
	Apit-R	AACCAGCAGCTTCCATTTGACG		
*A. nosocomialis*	Anos-F	GCCGCTCGTGAACGTGTAATC	394	
	Anos-R	CATCGTGTGGCATATCTTCAAC		
*A. lwoffii*	Alwo-F	CCGTGTCGGTCTGGTTCGTGTA	302	
	Alwo-R	CCGGCGTTTCAATTGGACATAC		
*A. ursingii*	Aurs-F	AGCGTGTCATCGTATCTCAG	319	
	Aurs-R	CCGCGTAAACGTTCAGGCACAAGA		
*A. haemolyticus*	Ahae-F	TGCACAGCAGGTAGTATCA	584	
	Ahae-R	GTAATTTCTTCTGCACCTAACTTA		
*A. baumannii*	Abau-F	CATAAACTGAGATAACTGGCTTGAACC	529	
	Abau-R	CGTCGACTTCACCTTTACCGTTAC		
*A. jhonsonii*	G24	CAACCAGCACCGAGGTATTT	244	[36]
	G25	TGAACAGGCGTAATTTGCAG		
*A. schindleri*	G60	TCGTATTTTCAGGCAAAGCTG	1090	
	G61	AAGCGCGTATCAAAAGGATG		
*A. bereziniae*	G30	GTTTGGCATTTTCAGGTTGTG	69	
	G31	TTAACGCAAATGCAGTCACC		

^1^ Primer set used for qPCR detection of *Acinetobacter* spp.

**Table 2:** GenBank accession numbers assigned to partial *16S rRNA* gene sequences obtained from *Acinetobacter* isolates in this study.

Isolate Identifier	GenBank Accession Number
524.2_LAM_UDLA_ECU 522_LAM_UDLA_ECU	PZ695520
	PZ695521
522.2_CTR_UDLA_ECU 390.1_CTR_UDLA_ECU	PZ695522
	PZ695523
98_CTR_UDLA_ECU 451.1_CTR_UDLA_ECU	PZ695524
	PZ695525
99_CTR_UDLA_ECU 93_CTR_UDLA_ECU	PZ695526
	PZ695527
97_CTR_UDLA_ECU 598.3_LAM_UDLA_ECU	PZ695528
	PZ695529
213.2_LAM_UDLA_ECU 327_LAM_UDLA_ECU	PZ695530
	PZ695531
276.2_LAM_UDLA_ECU 506.1_CTR_UDLA_ECU	PZ695532
	PZ695533
479.1_CTR_UDLA_ECU 74.3_LS_UDLA_ECU	PZ695534
	PZ695535
30.1_CTR_UDLA_ECU	PZ695536
524.2_LAM_UDLA_ECU 522_LAM_UDLA_ECU	PZ695520
	PZ695521
522.2_CTR_UDLA_ECU 390.1_CTR_UDLA_ECU	PZ695522
	PZ695523

**Table 3:** Molecular detection of *Acinetobacter* spp. by qPCR in cold-enriched raw milk samples according to province and producer size. Percentages in each row were calculated using the total number of samples analyzed within the corresponding province–producer size subgroup as the denominator. Percentages in the total row were calculated using all 400 samples.

Province	Producer Size	*Acinetobacter* spp.	Negative Samples	Total Samples
**PICHINCHA ^1^**	**Small**	73 (94.81%)	4 (5.19%)	77
	**Medium**	68 (95.77%)	3 (4.23%)	71
	**Large**	47 (90.38%)	5 (9.62%)	52
**MANABÍ**	**Small**	54 (77.14%)	16 (22.86%)	70
	**Medium**	49 (74.24%)	17 (25.76%)	66
	**Large**	51 (79.69%)	13 (20.31%)	64
**Total Positives**		**342** (**85.50%**)	**58** (**14.50%**)	**400**

^1^ Statistically significant difference.

## Data Availability

The partial *16S rRNA* gene sequences generated in this study have been deposited in the GenBank database under accession numbers PZ695520–PZ695536. The correspondence between isolate identifiers and accession numbers is provided in Table 2.

## Supplementary Materials

The following supporting information can be downloaded at: https://www.mdpi.com/article/10.3390/foods15152662/s1, Table S1: Complete Dataset; Table S2: Nucleotide Matrix; Table S3: Enriched raw milk samples selected for culture-based isolation according to their species-specific PCR.

## Abbreviations

The following abbreviations are used in this manuscript:

AGROCALIDAD	Agency for Phytosanitary and Zoosanitary Regulation and Control of Ecuador
ISO	International Organization for Standardization
INEN	Ecuadorian Standardization Service
INAMHI	National Institute of Meteorology and Hydrology
UCE	Universidad Central del Ecuador
UDLA	Universidad de las Americas
Ct	Cycle Threshold
spp.	Species pluralis
*A. lwoffi*	*Acinetobacter lwoffi*
*A. baumannii*	*Acinetobacter baumannii*
*A. calcoaceticus*	*Acinetobacter calcoaceticus*
*A. haemolyticus*	*Acinetobacter haemolyticus*
*A. pittii*	*Acinetobacter pittii*
*A. nosocomialis*	*Acinetobacter nosocomialis*
*A. ursingii*	*Acinetobacter ursingii*
*A. johnsonii*	*Acinetobacter johnsonii*
*A. schindleri*	*Acinetobacter schindleri*
*A. bereziniae*	*Acinetobacter bereziniae*
BHI	Brain Heart Infusion
DNA	Deoxyribonucleic Acid
TE buffer	Tris-EDTA buffer
GT	Guanidinium Thiocyanate
qPCR	Quantitative Polymerase Chain Reaction
PCR	Polymerase Chain Reaction
rRNA	Ribosomal Ribonucleic Acid
UDG	Uracil-DNA Glycosylase
UV	Ultraviolet
PMO	Pasteurized Milk Ordinance
OR	Odds Ratio
CI	Confidence Interval
NT	Nucleotide
BLAST	Basic Local Alignment Search Tool
LPSN	List of Prokaryotic names with Standing in Nomenclature
bp	Base pairs
