# Supplementary material for: Detection and Isolation of Psychrotrophic Acinetobacter spp. in Raw Milk from Ecuador: A Hidden Microbial Risk for Dairy Products
Source: Foods. 2026 Jul 29;15(15):2662. doi: 10.3390/foods15152662 (PMC13464793; doi:10.3390/foods15152662)
Supplement: Supplementary file 1 [file foods-15-02662-s001.zip › foods-4418116-supplementary.pdf]

Table S1 - Complete Dataset

Sample	Province	Producer Size	Climatic Period	Molecular Detection										
				qPCR	PCR									
				Acinetobacter spp.	*A. baumannii*	*A. haemolyticus*	*A. lwoffii*	*A. calcoaceticus*	*A. pittii*	*A. nosocomialis*	*A. ursingii*	*A. bereziniae*	*A. Jhonsoni*	*A. Schindleri*
3	PICHINCHA	MEDIUM	WARM	POSITIVE	NEGATIVE	NEGATIVE	POSITIVE	NEGATIVE	POSITIVE	NEGATIVE	NEGATIVE	NEGATIVE	POSITIVE	NEGATIVE
4	PICHINCHA	SMALL	WARM	POSITIVE	POSITIVE	NEGATIVE	NEGATIVE	NEGATIVE	NEGATIVE	POSITIVE	POSITIVE	NEGATIVE	POSITIVE	NEGATIVE
5	PICHINCHA	SMALL	WARM	POSITIVE	NEGATIVE	POSITIVE	POSITIVE	POSITIVE	NEGATIVE	NEGATIVE	NEGATIVE	NEGATIVE	POSITIVE	NEGATIVE
7	PICHINCHA	SMALL	WARM	POSITIVE	POSITIVE	NEGATIVE	POSITIVE	NEGATIVE	POSITIVE	NEGATIVE	POSITIVE	NEGATIVE	POSITIVE	NEGATIVE
8	PICHINCHA	SMALL	WARM	POSITIVE	NEGATIVE	POSITIVE	POSITIVE	NEGATIVE	NEGATIVE	NEGATIVE	NEGATIVE	NEGATIVE	POSITIVE	NEGATIVE
10	PICHINCHA	SMALL	WARM	POSITIVE	NEGATIVE	NEGATIVE	POSITIVE	NEGATIVE	POSITIVE	NEGATIVE	POSITIVE	NEGATIVE	POSITIVE	NEGATIVE
13	PICHINCHA	MEDIUM	WARM	POSITIVE	POSITIVE	NEGATIVE	NEGATIVE	NEGATIVE	POSITIVE	NEGATIVE	NEGATIVE	NEGATIVE	POSITIVE	NEGATIVE
14	PICHINCHA	SMALL	WARM	POSITIVE	POSITIVE	NEGATIVE	POSITIVE	NEGATIVE	NEGATIVE	NEGATIVE	POSITIVE	NEGATIVE	POSITIVE	NEGATIVE
15	PICHINCHA	SMALL	WARM	POSITIVE	NEGATIVE	NEGATIVE	POSITIVE	NEGATIVE	POSITIVE	NEGATIVE	POSITIVE	NEGATIVE	POSITIVE	NEGATIVE
16	PICHINCHA	MEDIUM	WARM	POSITIVE	POSITIVE	NEGATIVE	POSITIVE	NEGATIVE	POSITIVE	NEGATIVE	POSITIVE	NEGATIVE	POSITIVE	NEGATIVE
17	PICHINCHA	MEDIUM	WARM	POSITIVE	POSITIVE	NEGATIVE	POSITIVE	NEGATIVE	POSITIVE	NEGATIVE	POSITIVE	NEGATIVE	NEGATIVE	NEGATIVE
18	PICHINCHA	SMALL	WARM	POSITIVE	POSITIVE	NEGATIVE	NEGATIVE	NEGATIVE	NEGATIVE	POSITIVE	NEGATIVE	NEGATIVE	NEGATIVE	NEGATIVE
20	PICHINCHA	MEDIUM	WARM	POSITIVE	POSITIVE	NEGATIVE	POSITIVE	NEGATIVE	POSITIVE	NEGATIVE	NEGATIVE	NEGATIVE	POSITIVE	NEGATIVE
21	PICHINCHA	SMALL	WARM	POSITIVE	POSITIVE	NEGATIVE	POSITIVE	NEGATIVE	POSITIVE	NEGATIVE	NEGATIVE	NEGATIVE	POSITIVE	NEGATIVE
24	PICHINCHA	MEDIUM	WARM	POSITIVE	POSITIVE	NEGATIVE	NEGATIVE	NEGATIVE	POSITIVE	POSITIVE	POSITIVE	NEGATIVE	POSITIVE	NEGATIVE
26	PICHINCHA	LARGE	WARM	POSITIVE	POSITIVE	NEGATIVE	POSITIVE	NEGATIVE	POSITIVE	POSITIVE	POSITIVE	NEGATIVE	POSITIVE	NEGATIVE
29	PICHINCHA	MEDIUM	WARM	POSITIVE	POSITIVE	POSITIVE	POSITIVE	NEGATIVE	NEGATIVE	NEGATIVE	NEGATIVE	NEGATIVE	POSITIVE	NEGATIVE
33	PICHINCHA	SMALL	WARM	POSITIVE	POSITIVE	NEGATIVE	POSITIVE	NEGATIVE	NEGATIVE	NEGATIVE	POSITIVE	NEGATIVE	POSITIVE	NEGATIVE
38	PICHINCHA	LARGE	WARM	POSITIVE	POSITIVE	NEGATIVE	POSITIVE	NEGATIVE	POSITIVE	POSITIVE	POSITIVE	NEGATIVE	POSITIVE	NEGATIVE
39	PICHINCHA	SMALL	WARM	POSITIVE	POSITIVE	NEGATIVE	NEGATIVE	NEGATIVE	POSITIVE	POSITIVE	POSITIVE	NEGATIVE	POSITIVE	NEGATIVE
40	PICHINCHA	SMALL	WARM	POSITIVE	POSITIVE	NEGATIVE	POSITIVE	NEGATIVE	NEGATIVE	NEGATIVE	NEGATIVE	NEGATIVE	POSITIVE	NEGATIVE
42	PICHINCHA	SMALL	WARM	POSITIVE	POSITIVE	NEGATIVE	POSITIVE	NEGATIVE	POSITIVE	NEGATIVE	NEGATIVE	NEGATIVE	POSITIVE	NEGATIVE
43	PICHINCHA	SMALL	WARM	POSITIVE	POSITIVE	NEGATIVE	NEGATIVE	NEGATIVE	NEGATIVE	NEGATIVE	POSITIVE	NEGATIVE	POSITIVE	NEGATIVE
44	PICHINCHA	SMALL	WARM	POSITIVE	POSITIVE	NEGATIVE	POSITIVE	NEGATIVE	NEGATIVE	NEGATIVE	NEGATIVE	NEGATIVE	POSITIVE	NEGATIVE
46	PICHINCHA	MEDIUM	RAINY	POSITIVE	POSITIVE	NEGATIVE	POSITIVE	NEGATIVE	NEGATIVE	NEGATIVE	NEGATIVE	NEGATIVE	NEGATIVE	NEGATIVE
52	PICHINCHA	SMALL	RAINY	POSITIVE	POSITIVE	NEGATIVE	POSITIVE	NEGATIVE	NEGATIVE	NEGATIVE	NEGATIVE	NEGATIVE	POSITIVE	NEGATIVE
53	PICHINCHA	MEDIUM	RAINY	POSITIVE	POSITIVE	NEGATIVE	POSITIVE	NEGATIVE	NEGATIVE	NEGATIVE	NEGATIVE	NEGATIVE	POSITIVE	NEGATIVE
54	PICHINCHA	LARGE	RAINY	POSITIVE	POSITIVE	NEGATIVE	POSITIVE	NEGATIVE	NEGATIVE	NEGATIVE	NEGATIVE	NEGATIVE	POSITIVE	NEGATIVE
55	PICHINCHA	MEDIUM	RAINY	POSITIVE	POSITIVE	NEGATIVE	POSITIVE	NEGATIVE	POSITIVE	NEGATIVE	NEGATIVE	NEGATIVE	POSITIVE	NEGATIVE
57	PICHINCHA	MEDIUM	RAINY	POSITIVE	POSITIVE	NEGATIVE	POSITIVE	NEGATIVE	POSITIVE	NEGATIVE	NEGATIVE	NEGATIVE	POSITIVE	NEGATIVE
58	PICHINCHA	MEDIUM	RAINY	POSITIVE	POSITIVE	NEGATIVE	POSITIVE	NEGATIVE	POSITIVE	NEGATIVE	NEGATIVE	NEGATIVE	POSITIVE	NEGATIVE
60	PICHINCHA	MEDIUM	RAINY	POSITIVE	POSITIVE	NEGATIVE	POSITIVE	NEGATIVE	POSITIVE	NEGATIVE	NEGATIVE	POSITIVE	POSITIVE	POSITIVE
61	PICHINCHA	SMALL	RAINY	POSITIVE	POSITIVE	NEGATIVE	POSITIVE	NEGATIVE	NEGATIVE	NEGATIVE	NEGATIVE	NEGATIVE	NEGATIVE	NEGATIVE
62	PICHINCHA	LARGE	RAINY	POSITIVE	POSITIVE	NEGATIVE	POSITIVE	NEGATIVE	NEGATIVE	POSITIVE	NEGATIVE	NEGATIVE	POSITIVE	NEGATIVE
63	PICHINCHA	MEDIUM	RAINY	POSITIVE	POSITIVE	NEGATIVE	POSITIVE	NEGATIVE	POSITIVE	POSITIVE	NEGATIVE	NEGATIVE	NEGATIVE	NEGATIVE
65	PICHINCHA	MEDIUM	RAINY	POSITIVE	POSITIVE	NEGATIVE	POSITIVE	NEGATIVE	POSITIVE	NEGATIVE	NEGATIVE	NEGATIVE	POSITIVE	NEGATIVE
69	MANABÍ	LARGE	RAINY	POSITIVE	POSITIVE	NEGATIVE	POSITIVE	NEGATIVE	NEGATIVE	NEGATIVE	NEGATIVE	NEGATIVE	NEGATIVE	NEGATIVE
71	MANABÍ	LARGE	RAINY	POSITIVE	POSITIVE	NEGATIVE	POSITIVE	NEGATIVE	POSITIVE	NEGATIVE	NEGATIVE	NEGATIVE	POSITIVE	NEGATIVE
74	MANABÍ	SMALL	RAINY	POSITIVE	POSITIVE	NEGATIVE	POSITIVE	NEGATIVE	POSITIVE	POSITIVE	POSITIVE	NEGATIVE	POSITIVE	POSITIVE
75	MANABÍ	LARGE	RAINY	POSITIVE	POSITIVE	NEGATIVE	POSITIVE	NEGATIVE	POSITIVE	NEGATIVE	NEGATIVE	NEGATIVE	NEGATIVE	NEGATIVE
78	PICHINCHA	MEDIUM	RAINY	POSITIVE	POSITIVE	NEGATIVE	POSITIVE	NEGATIVE	POSITIVE	NEGATIVE	POSITIVE	NEGATIVE	POSITIVE	POSITIVE
79	PICHINCHA	LARGE	RAINY	POSITIVE	POSITIVE	NEGATIVE	POSITIVE	NEGATIVE	POSITIVE	POSITIVE	NEGATIVE	POSITIVE	POSITIVE	POSITIVE
81	PICHINCHA	SMALL	RAINY	POSITIVE	POSITIVE	NEGATIVE	POSITIVE	NEGATIVE	NEGATIVE	POSITIVE	NEGATIVE	NEGATIVE	NEGATIVE	NEGATIVE
83	PICHINCHA	MEDIUM	RAINY	POSITIVE	POSITIVE	NEGATIVE	POSITIVE	NEGATIVE	NEGATIVE	NEGATIVE	NEGATIVE	NEGATIVE	NEGATIVE	NEGATIVE
92	MANABÍ	LARGE	RAINY	POSITIVE	POSITIVE	NEGATIVE	POSITIVE	NEGATIVE	POSITIVE	NEGATIVE	NEGATIVE	NEGATIVE	POSITIVE	NEGATIVE
93	MANABÍ	SMALL	RAINY	POSITIVE	POSITIVE	NEGATIVE	POSITIVE	NEGATIVE	POSITIVE	NEGATIVE	NEGATIVE	NEGATIVE	POSITIVE	NEGATIVE
94	MANABÍ	SMALL	RAINY	POSITIVE	POSITIVE	NEGATIVE	POSITIVE	NEGATIVE	POSITIVE	NEGATIVE	NEGATIVE	NEGATIVE	POSITIVE	NEGATIVE
97	MANABÍ	MEDIUM	RAINY	POSITIVE	POSITIVE	NEGATIVE	POSITIVE	NEGATIVE	POSITIVE	POSITIVE	NEGATIVE	NEGATIVE	POSITIVE	NEGATIVE
99	MANABÍ	LARGE	RAINY	POSITIVE	POSITIVE	NEGATIVE	POSITIVE	POSITIVE	POSITIVE	NEGATIVE	NEGATIVE	NEGATIVE	POSITIVE	NEGATIVE
103	MANABÍ	SMALL	RAINY	POSITIVE	POSITIVE	POSITIVE	POSITIVE	NEGATIVE	POSITIVE	NEGATIVE	NEGATIVE	NEGATIVE	POSITIVE	NEGATIVE
104	MANABÍ	SMALL	RAINY	POSITIVE	POSITIVE	NEGATIVE	POSITIVE	NEGATIVE	POSITIVE	NEGATIVE	NEGATIVE	NEGATIVE	POSITIVE	NEGATIVE
105	MANABÍ	LARGE	RAINY	POSITIVE	POSITIVE	NEGATIVE	POSITIVE	POSITIVE	POSITIVE	NEGATIVE	NEGATIVE	NEGATIVE	POSITIVE	NEGATIVE
106	MANABÍ	SMALL	RAINY	POSITIVE	POSITIVE	NEGATIVE	NEGATIVE	NEGATIVE	POSITIVE	NEGATIVE	NEGATIVE	NEGATIVE	NEGATIVE	NEGATIVE
107	MANABÍ	MEDIUM	RAINY	POSITIVE	POSITIVE	NEGATIVE	POSITIVE	POSITIVE	POSITIVE	NEGATIVE	NEGATIVE	NEGATIVE	NEGATIVE	NEGATIVE
108	MANABÍ	LARGE	RAINY	POSITIVE	NEGATIVE	NEGATIVE	POSITIVE	NEGATIVE	NEGATIVE	NEGATIVE	POSITIVE	NEGATIVE	POSITIVE	NEGATIVE
110	PICHINCHA	SMALL	RAINY	POSITIVE	POSITIVE	NEGATIVE	NEGATIVE	NEGATIVE	NEGATIVE	NEGATIVE	POSITIVE	NEGATIVE	NEGATIVE	NEGATIVE
111	PICHINCHA	SMALL	RAINY	POSITIVE	NEGATIVE	NEGATIVE	NEGATIVE	POSITIVE	NEGATIVE	NEGATIVE	NEGATIVE	NEGATIVE	POSITIVE	NEGATIVE
113	PICHINCHA	SMALL	RAINY	POSITIVE	POSITIVE	NEGATIVE	POSITIVE	NEGATIVE	POSITIVE	NEGATIVE	POSITIVE	NEGATIVE	NEGATIVE	NEGATIVE
114	PICHINCHA	MEDIUM	RAINY	POSITIVE	POSITIVE	NEGATIVE	POSITIVE	NEGATIVE	POSITIVE	NEGATIVE	NEGATIVE	NEGATIVE	POSITIVE	NEGATIVE
116	PICHINCHA	MEDIUM	RAINY	POSITIVE	POSITIVE	NEGATIVE	NEGATIVE	NEGATIVE	NEGATIVE	NEGATIVE	NEGATIVE	NEGATIVE	NEGATIVE	NEGATIVE
117	PICHINCHA	SMALL	RAINY	POSITIVE	NEGATIVE	NEGATIVE	POSITIVE	NEGATIVE	NEGATIVE	NEGATIVE	NEGATIVE	NEGATIVE	NEGATIVE	NEGATIVE
118	PICHINCHA	MEDIUM	RAINY	POSITIVE	POSITIVE	NEGATIVE	POSITIVE	POSITIVE	POSITIVE	NEGATIVE	NEGATIVE	NEGATIVE	NEGATIVE	NEGATIVE
120	PICHINCHA	LARGE	RAINY	POSITIVE	NEGATIVE	NEGATIVE	POSITIVE	POSITIVE	NEGATIVE	NEGATIVE	NEGATIVE	NEGATIVE	POSITIVE	NEGATIVE
122	PICHINCHA	LARGE	RAINY	POSITIVE	NEGATIVE	NEGATIVE	POSITIVE	POSITIVE	POSITIVE	NEGATIVE	NEGATIVE	POSITIVE	POSITIVE	NEGATIVE
124	PICHINCHA	SMALL	RAINY	POSITIVE	POSITIVE	NEGATIVE	POSITIVE	NEGATIVE	POSITIVE	POSITIVE	NEGATIVE	NEGATIVE	POSITIVE	NEGATIVE
126	PICHINCHA	LARGE	RAINY	POSITIVE	POSITIVE	NEGATIVE	POSITIVE	NEGATIVE	POSITIVE	NEGATIVE	NEGATIVE	NEGATIVE	POSITIVE	NEGATIVE
127	PICHINCHA	LARGE	RAINY	POSITIVE	POSITIVE	NEGATIVE	POSITIVE	NEGATIVE	NEGATIVE	NEGATIVE	NEGATIVE	NEGATIVE	POSITIVE	NEGATIVE
128	PICHINCHA	SMALL	RAINY	POSITIVE	POSITIVE	NEGATIVE	NEGATIVE	NEGATIVE	POSITIVE	POSITIVE	NEGATIVE	NEGATIVE	NEGATIVE	NEGATIVE
129	PICHINCHA	SMALL	RAINY	POSITIVE	NEGATIVE	NEGATIVE	POSITIVE	NEGATIVE	POSITIVE	POSITIVE	NEGATIVE	NEGATIVE	POSITIVE	NEGATIVE
130	PICHINCHA	LARGE	RAINY	POSITIVE	POSITIVE	NEGATIVE	POSITIVE	POSITIVE	POSITIVE	NEGATIVE	POSITIVE	NEGATIVE	POSITIVE	NEGATIVE
131	PICHINCHA	MEDIUM	RAINY	POSITIVE	POSITIVE	NEGATIVE	POSITIVE	POSITIVE	POSITIVE	NEGATIVE	POSITIVE	NEGATIVE	POSITIVE	NEGATIVE

132	PICHINCHA	LARGE	RAINY	POSITIVE	POSITIVE	NEGATIVE	POSITIVE	NEGATIVE	NEGATIVE	NEGATIVE	POSITIVE	NEGATIVE	POSITIVE	NEGATIVE
133	PICHINCHA	LARGE	RAINY	POSITIVE	POSITIVE	NEGATIVE	POSITIVE	NEGATIVE	NEGATIVE	NEGATIVE	NEGATIVE	NEGATIVE	POSITIVE	NEGATIVE
134	PICHINCHA	SMALL	RAINY	POSITIVE	POSITIVE	NEGATIVE	POSITIVE	NEGATIVE	NEGATIVE	NEGATIVE	POSITIVE	NEGATIVE	POSITIVE	NEGATIVE
135	PICHINCHA	LARGE	RAINY	POSITIVE	POSITIVE	NEGATIVE	POSITIVE	NEGATIVE	POSITIVE	NEGATIVE	NEGATIVE	NEGATIVE	POSITIVE	NEGATIVE
137	PICHINCHA	LARGE	RAINY	POSITIVE	POSITIVE	NEGATIVE	POSITIVE	NEGATIVE	POSITIVE	NEGATIVE	POSITIVE	NEGATIVE	POSITIVE	NEGATIVE
139	PICHINCHA	LARGE	RAINY	POSITIVE	POSITIVE	NEGATIVE	POSITIVE	POSITIVE	POSITIVE	NEGATIVE	POSITIVE	NEGATIVE	POSITIVE	NEGATIVE
140	PICHINCHA	MEDIUM	RAINY	POSITIVE	POSITIVE	NEGATIVE	POSITIVE	NEGATIVE	POSITIVE	NEGATIVE	POSITIVE	NEGATIVE	POSITIVE	NEGATIVE
143	PICHINCHA	LARGE	RAINY	POSITIVE	POSITIVE	NEGATIVE	POSITIVE	POSITIVE	NEGATIVE	NEGATIVE	NEGATIVE	NEGATIVE	POSITIVE	NEGATIVE
144	PICHINCHA	MEDIUM	RAINY	POSITIVE	POSITIVE	NEGATIVE	POSITIVE	NEGATIVE	POSITIVE	NEGATIVE	POSITIVE	NEGATIVE	POSITIVE	NEGATIVE
146	PICHINCHA	LARGE	RAINY	POSITIVE	POSITIVE	POSITIVE	POSITIVE	NEGATIVE	POSITIVE	NEGATIVE	POSITIVE	NEGATIVE	POSITIVE	NEGATIVE
147	PICHINCHA	MEDIUM	RAINY	POSITIVE	POSITIVE	NEGATIVE	POSITIVE	NEGATIVE	POSITIVE	POSITIVE	POSITIVE	NEGATIVE	POSITIVE	NEGATIVE
148	PICHINCHA	SMALL	RAINY	POSITIVE	POSITIVE	NEGATIVE	POSITIVE	NEGATIVE	NEGATIVE	NEGATIVE	POSITIVE	NEGATIVE	POSITIVE	NEGATIVE
150	PICHINCHA	LARGE	RAINY	POSITIVE	POSITIVE	NEGATIVE	POSITIVE	NEGATIVE	POSITIVE	NEGATIVE	POSITIVE	NEGATIVE	POSITIVE	NEGATIVE
151	PICHINCHA	MEDIUM	RAINY	POSITIVE	POSITIVE	NEGATIVE	POSITIVE	NEGATIVE	NEGATIVE	NEGATIVE	POSITIVE	NEGATIVE	POSITIVE	NEGATIVE
152	PICHINCHA	SMALL	RAINY	POSITIVE	POSITIVE	POSITIVE	POSITIVE	POSITIVE	POSITIVE	NEGATIVE	POSITIVE	NEGATIVE	POSITIVE	NEGATIVE
154	PICHINCHA	LARGE	RAINY	POSITIVE	POSITIVE	NEGATIVE	NEGATIVE	POSITIVE	NEGATIVE	NEGATIVE	NEGATIVE	NEGATIVE	POSITIVE	NEGATIVE
155	PICHINCHA	LARGE	RAINY	POSITIVE	POSITIVE	NEGATIVE	POSITIVE	NEGATIVE	NEGATIVE	NEGATIVE	NEGATIVE	NEGATIVE	POSITIVE	NEGATIVE
157	PICHINCHA	SMALL	RAINY	POSITIVE	POSITIVE	NEGATIVE	NEGATIVE	NEGATIVE	NEGATIVE	NEGATIVE	NEGATIVE	NEGATIVE	NEGATIVE	NEGATIVE
158	PICHINCHA	MEDIUM	RAINY	POSITIVE	POSITIVE	NEGATIVE	NEGATIVE	POSITIVE	POSITIVE	NEGATIVE	NEGATIVE	NEGATIVE	POSITIVE	NEGATIVE
159	PICHINCHA	MEDIUM	RAINY	POSITIVE	POSITIVE	NEGATIVE	NEGATIVE	NEGATIVE	NEGATIVE	NEGATIVE	NEGATIVE	NEGATIVE	NEGATIVE	NEGATIVE
160	PICHINCHA	MEDIUM	RAINY	POSITIVE	POSITIVE	NEGATIVE	NEGATIVE	NEGATIVE	POSITIVE	NEGATIVE	NEGATIVE	NEGATIVE	NEGATIVE	NEGATIVE
161	PICHINCHA	SMALL	RAINY	POSITIVE	POSITIVE	NEGATIVE	NEGATIVE	POSITIVE	POSITIVE	NEGATIVE	NEGATIVE	NEGATIVE	POSITIVE	NEGATIVE
162	PICHINCHA	MEDIUM	RAINY	POSITIVE	POSITIVE	NEGATIVE	NEGATIVE	NEGATIVE	NEGATIVE	NEGATIVE	NEGATIVE	NEGATIVE	NEGATIVE	NEGATIVE
163	PICHINCHA	MEDIUM	RAINY	POSITIVE	POSITIVE	NEGATIVE	NEGATIVE	NEGATIVE	POSITIVE	NEGATIVE	NEGATIVE	NEGATIVE	POSITIVE	NEGATIVE
165	PICHINCHA	MEDIUM	RAINY	POSITIVE	POSITIVE	NEGATIVE	NEGATIVE	NEGATIVE	POSITIVE	NEGATIVE	NEGATIVE	NEGATIVE	NEGATIVE	NEGATIVE
166	PICHINCHA	MEDIUM	RAINY	POSITIVE	POSITIVE	NEGATIVE	POSITIVE	NEGATIVE	NEGATIVE	POSITIVE	NEGATIVE	NEGATIVE	NEGATIVE	NEGATIVE
167	PICHINCHA	MEDIUM	RAINY	POSITIVE	POSITIVE	NEGATIVE	NEGATIVE	NEGATIVE	POSITIVE	NEGATIVE	NEGATIVE	NEGATIVE	POSITIVE	NEGATIVE
168	PICHINCHA	SMALL	RAINY	POSITIVE	POSITIVE	NEGATIVE	NEGATIVE	NEGATIVE	POSITIVE	NEGATIVE	NEGATIVE	NEGATIVE	NEGATIVE	NEGATIVE
169	PICHINCHA	SMALL	RAINY	POSITIVE	POSITIVE	NEGATIVE	POSITIVE	NEGATIVE	POSITIVE	NEGATIVE	NEGATIVE	NEGATIVE	POSITIVE	NEGATIVE
170	PICHINCHA	MEDIUM	RAINY	POSITIVE	POSITIVE	NEGATIVE	NEGATIVE	NEGATIVE	NEGATIVE	NEGATIVE	NEGATIVE	NEGATIVE	NEGATIVE	NEGATIVE
171	PICHINCHA	MEDIUM	RAINY	POSITIVE	NEGATIVE	NEGATIVE	NEGATIVE	POSITIVE	NEGATIVE	NEGATIVE	NEGATIVE	NEGATIVE	NEGATIVE	NEGATIVE
172	PICHINCHA	SMALL	RAINY	POSITIVE	POSITIVE	NEGATIVE	POSITIVE	NEGATIVE	NEGATIVE	NEGATIVE	NEGATIVE	NEGATIVE	POSITIVE	NEGATIVE
173	PICHINCHA	MEDIUM	RAINY	POSITIVE	POSITIVE	NEGATIVE	POSITIVE	NEGATIVE	NEGATIVE	NEGATIVE	NEGATIVE	NEGATIVE	NEGATIVE	NEGATIVE
174	PICHINCHA	LARGE	RAINY	POSITIVE	POSITIVE	NEGATIVE	POSITIVE	NEGATIVE	NEGATIVE	NEGATIVE	NEGATIVE	NEGATIVE	NEGATIVE	NEGATIVE
175	PICHINCHA	SMALL	RAINY	POSITIVE	POSITIVE	NEGATIVE	NEGATIVE	NEGATIVE	NEGATIVE	NEGATIVE	NEGATIVE	NEGATIVE	NEGATIVE	NEGATIVE
176	PICHINCHA	MEDIUM	RAINY	POSITIVE	POSITIVE	NEGATIVE	POSITIVE	NEGATIVE	POSITIVE	NEGATIVE	NEGATIVE	NEGATIVE	POSITIVE	NEGATIVE
177	PICHINCHA	SMALL	RAINY	POSITIVE	POSITIVE	NEGATIVE	NEGATIVE	NEGATIVE	POSITIVE	POSITIVE	NEGATIVE	NEGATIVE	NEGATIVE	NEGATIVE
178	PICHINCHA	LARGE	RAINY	POSITIVE	POSITIVE	NEGATIVE	POSITIVE	NEGATIVE	NEGATIVE	NEGATIVE	NEGATIVE	NEGATIVE	POSITIVE	POSITIVE
179	PICHINCHA	MEDIUM	RAINY	POSITIVE	POSITIVE	NEGATIVE	POSITIVE	NEGATIVE	POSITIVE	NEGATIVE	NEGATIVE	NEGATIVE	POSITIVE	NEGATIVE
182	PICHINCHA	MEDIUM	RAINY	POSITIVE	POSITIVE	NEGATIVE	POSITIVE	NEGATIVE	NEGATIVE	NEGATIVE	NEGATIVE	NEGATIVE	POSITIVE	NEGATIVE
183	PICHINCHA	MEDIUM	RAINY	POSITIVE	POSITIVE	NEGATIVE	POSITIVE	NEGATIVE	NEGATIVE	NEGATIVE	NEGATIVE	POSITIVE	POSITIVE	NEGATIVE
185	PICHINCHA	MEDIUM	RAINY	POSITIVE	POSITIVE	NEGATIVE	POSITIVE	NEGATIVE	POSITIVE	POSITIVE	NEGATIVE	NEGATIVE	NEGATIVE	NEGATIVE
186	PICHINCHA	LARGE	RAINY	POSITIVE	POSITIVE	NEGATIVE	POSITIVE	NEGATIVE	NEGATIVE	NEGATIVE	NEGATIVE	POSITIVE	POSITIVE	POSITIVE
187	PICHINCHA	LARGE	RAINY	POSITIVE	POSITIVE	NEGATIVE	POSITIVE	NEGATIVE	NEGATIVE	NEGATIVE	NEGATIVE	NEGATIVE	POSITIVE	NEGATIVE
189	PICHINCHA	LARGE	RAINY	POSITIVE	POSITIVE	NEGATIVE	POSITIVE	POSITIVE	NEGATIVE	NEGATIVE	NEGATIVE	NEGATIVE	POSITIVE	NEGATIVE
191	PICHINCHA	LARGE	RAINY	POSITIVE	POSITIVE	NEGATIVE	POSITIVE	POSITIVE	POSITIVE	NEGATIVE	NEGATIVE	NEGATIVE	NEGATIVE	NEGATIVE
192	PICHINCHA	MEDIUM	RAINY	POSITIVE	POSITIVE	NEGATIVE	POSITIVE	NEGATIVE	POSITIVE	POSITIVE	NEGATIVE	POSITIVE	POSITIVE	NEGATIVE
193	PICHINCHA	LARGE	RAINY	POSITIVE	POSITIVE	NEGATIVE	POSITIVE	NEGATIVE	NEGATIVE	POSITIVE	NEGATIVE	POSITIVE	NEGATIVE	POSITIVE
195	MANABÍ	MEDIUM	RAINY	POSITIVE	POSITIVE	NEGATIVE	POSITIVE	NEGATIVE	NEGATIVE	NEGATIVE	NEGATIVE	NEGATIVE	NEGATIVE	NEGATIVE
197	MANABÍ	MEDIUM	RAINY	POSITIVE	POSITIVE	NEGATIVE	POSITIVE	NEGATIVE	NEGATIVE	NEGATIVE	POSITIVE	NEGATIVE	NEGATIVE	NEGATIVE
198	MANABÍ	SMALL	RAINY	POSITIVE	POSITIVE	NEGATIVE	POSITIVE	NEGATIVE	POSITIVE	NEGATIVE	POSITIVE	NEGATIVE	POSITIVE	NEGATIVE
199	MANABÍ	LARGE	RAINY	POSITIVE	POSITIVE	NEGATIVE	NEGATIVE	NEGATIVE	NEGATIVE	POSITIVE	POSITIVE	NEGATIVE	POSITIVE	NEGATIVE
200	MANABÍ	SMALL	RAINY	POSITIVE	POSITIVE	NEGATIVE	NEGATIVE	NEGATIVE	NEGATIVE	NEGATIVE	POSITIVE	NEGATIVE	NEGATIVE	NEGATIVE
201	MANABÍ	MEDIUM	RAINY	POSITIVE	POSITIVE	NEGATIVE	NEGATIVE	NEGATIVE	POSITIVE	NEGATIVE	POSITIVE	NEGATIVE	NEGATIVE	NEGATIVE
204	MANABÍ	LARGE	RAINY	POSITIVE	NEGATIVE	NEGATIVE	POSITIVE	POSITIVE	POSITIVE	POSITIVE	POSITIVE	NEGATIVE	NEGATIVE	NEGATIVE
206	MANABÍ	LARGE	RAINY	POSITIVE	NEGATIVE	NEGATIVE	POSITIVE	NEGATIVE	POSITIVE	POSITIVE	POSITIVE	NEGATIVE	POSITIVE	NEGATIVE
208	MANABÍ	MEDIUM	RAINY	POSITIVE	NEGATIVE	NEGATIVE	POSITIVE	NEGATIVE	NEGATIVE	NEGATIVE	POSITIVE	NEGATIVE	POSITIVE	NEGATIVE
209	MANABÍ	SMALL	RAINY	POSITIVE	NEGATIVE	NEGATIVE	POSITIVE	NEGATIVE	NEGATIVE	POSITIVE	POSITIVE	NEGATIVE	NEGATIVE	NEGATIVE
210	MANABÍ	MEDIUM	RAINY	POSITIVE	NEGATIVE	NEGATIVE	POSITIVE	NEGATIVE	NEGATIVE	NEGATIVE	POSITIVE	NEGATIVE	NEGATIVE	NEGATIVE
211	MANABÍ	LARGE	RAINY	POSITIVE	NEGATIVE	NEGATIVE	POSITIVE	NEGATIVE	NEGATIVE	NEGATIVE	POSITIVE	NEGATIVE	POSITIVE	NEGATIVE
212	MANABÍ	SMALL	RAINY	POSITIVE	NEGATIVE	NEGATIVE	POSITIVE	NEGATIVE	NEGATIVE	NEGATIVE	NEGATIVE	NEGATIVE	POSITIVE	NEGATIVE
215	MANABÍ	MEDIUM	RAINY	POSITIVE	NEGATIVE	NEGATIVE	POSITIVE	NEGATIVE	NEGATIVE	POSITIVE	POSITIVE	NEGATIVE	POSITIVE	NEGATIVE
217	MANABÍ	LARGE	RAINY	POSITIVE	NEGATIVE	NEGATIVE	POSITIVE	NEGATIVE	POSITIVE	POSITIVE	POSITIVE	POSITIVE	POSITIVE	POSITIVE
218	MANABÍ	SMALL	RAINY	POSITIVE	NEGATIVE	NEGATIVE	POSITIVE	NEGATIVE	POSITIVE	POSITIVE	POSITIVE	NEGATIVE	POSITIVE	POSITIVE
220	MANABÍ	MEDIUM	RAINY	POSITIVE	NEGATIVE	NEGATIVE	POSITIVE	NEGATIVE	NEGATIVE	NEGATIVE	POSITIVE	POSITIVE	POSITIVE	POSITIVE
221	MANABÍ	SMALL	RAINY	POSITIVE	NEGATIVE	NEGATIVE	POSITIVE	NEGATIVE	NEGATIVE	POSITIVE	POSITIVE	POSITIVE	POSITIVE	POSITIVE
222	MANABÍ	LARGE	RAINY	POSITIVE	NEGATIVE	NEGATIVE	POSITIVE	NEGATIVE	POSITIVE	POSITIVE	POSITIVE	NEGATIVE	POSITIVE	NEGATIVE
223	MANABÍ	LARGE	RAINY	POSITIVE	NEGATIVE	NEGATIVE	POSITIVE	NEGATIVE	POSITIVE	POSITIVE	POSITIVE	POSITIVE	POSITIVE	POSITIVE
224	MANABÍ	LARGE	RAINY	POSITIVE	NEGATIVE	NEGATIVE	POSITIVE	NEGATIVE	POSITIVE	POSITIVE	POSITIVE	NEGATIVE	POSITIVE	NEGATIVE
228	MANABÍ	MEDIUM	RAINY	POSITIVE	NEGATIVE	NEGATIVE	POSITIVE	NEGATIVE	POSITIVE	POSITIVE	POSITIVE	POSITIVE	POSITIVE	POSITIVE
230	MANABÍ	MEDIUM	RAINY	POSITIVE	NEGATIVE	NEGATIVE	POSITIVE	NEGATIVE	POSITIVE	POSITIVE	POSITIVE	NEGATIVE	POSITIVE	POSITIVE
231	MANABÍ	SMALL	RAINY	POSITIVE	NEGATIVE	NEGATIVE	POSITIVE	NEGATIVE	NEGATIVE	POSITIVE	POSITIVE	NEGATIVE	POSITIVE	NEGATIVE
232	MANABÍ	SMALL	RAINY	POSITIVE	NEGATIVE	NEGATIVE	POSITIVE	NEGATIVE	NEGATIVE	NEGATIVE	POSITIVE	NEGATIVE	NEGATIVE	NEGATIVE
233	MANABÍ	MEDIUM	RAINY	POSITIVE	NEGATIVE	NEGATIVE	POSITIVE	NEGATIVE	POSITIVE	POSITIVE	POSITIVE	NEGATIVE	POSITIVE	POSITIVE

234	MANABÍ	LARGE	WARM	POSITIVE	NEGATIVE	POSITIVE	POSITIVE	NEGATIVE	POSITIVE	POSITIVE	POSITIVE	NEGATIVE	POSITIVE	NEGATIVE
235	MANABÍ	MEDIUM	WARM	POSITIVE	NEGATIVE	NEGATIVE	POSITIVE	NEGATIVE	POSITIVE	POSITIVE	POSITIVE	NEGATIVE	NEGATIVE	NEGATIVE
236	MANABÍ	MEDIUM	WARM	POSITIVE	NEGATIVE	NEGATIVE	POSITIVE	NEGATIVE	POSITIVE	POSITIVE	POSITIVE	POSITIVE	NEGATIVE	NEGATIVE
239	MANABÍ	LARGE	WARM	POSITIVE	NEGATIVE	NEGATIVE	POSITIVE	NEGATIVE	POSITIVE	POSITIVE	POSITIVE	NEGATIVE	POSITIVE	NEGATIVE
242	MANABÍ	MEDIUM	WARM	POSITIVE	NEGATIVE	NEGATIVE	POSITIVE	NEGATIVE	POSITIVE	POSITIVE	POSITIVE	POSITIVE	POSITIVE	NEGATIVE
243	MANABÍ	LARGE	WARM	POSITIVE	NEGATIVE	NEGATIVE	POSITIVE	NEGATIVE	POSITIVE	POSITIVE	POSITIVE	NEGATIVE	NEGATIVE	NEGATIVE
244	MANABÍ	SMALL	WARM	POSITIVE	NEGATIVE	NEGATIVE	POSITIVE	NEGATIVE	POSITIVE	POSITIVE	NEGATIVE	NEGATIVE	POSITIVE	NEGATIVE
246	MANABÍ	SMALL	WARM	POSITIVE	NEGATIVE	NEGATIVE	POSITIVE	NEGATIVE	NEGATIVE	NEGATIVE	POSITIVE	NEGATIVE	NEGATIVE	NEGATIVE
247	MANABÍ	LARGE	WARM	POSITIVE	NEGATIVE	NEGATIVE	POSITIVE	NEGATIVE	POSITIVE	NEGATIVE	POSITIVE	NEGATIVE	POSITIVE	NEGATIVE
248	MANABÍ	MEDIUM	WARM	POSITIVE	NEGATIVE	NEGATIVE	POSITIVE	NEGATIVE	POSITIVE	NEGATIVE	POSITIVE	NEGATIVE	NEGATIVE	NEGATIVE
252	MANABÍ	MEDIUM	WARM	POSITIVE	NEGATIVE	NEGATIVE	POSITIVE	NEGATIVE	POSITIVE	NEGATIVE	POSITIVE	NEGATIVE	NEGATIVE	NEGATIVE
256	MANABÍ	MEDIUM	WARM	POSITIVE	NEGATIVE	NEGATIVE	POSITIVE	NEGATIVE	POSITIVE	POSITIVE	POSITIVE	NEGATIVE	NEGATIVE	NEGATIVE
258	MANABÍ	SMALL	WARM	POSITIVE	NEGATIVE	NEGATIVE	POSITIVE	NEGATIVE	NEGATIVE	POSITIVE	POSITIVE	NEGATIVE	NEGATIVE	NEGATIVE
262	MANABÍ	SMALL	WARM	POSITIVE	NEGATIVE	NEGATIVE	POSITIVE	NEGATIVE	POSITIVE	POSITIVE	POSITIVE	NEGATIVE	NEGATIVE	NEGATIVE
267	MANABÍ	LARGE	WARM	POSITIVE	NEGATIVE	NEGATIVE	POSITIVE	NEGATIVE	NEGATIVE	POSITIVE	POSITIVE	NEGATIVE	POSITIVE	NEGATIVE
268	MANABÍ	SMALL	WARM	POSITIVE	NEGATIVE	NEGATIVE	POSITIVE	NEGATIVE	POSITIVE	POSITIVE	POSITIVE	NEGATIVE	POSITIVE	NEGATIVE
269	MANABÍ	MEDIUM	WARM	POSITIVE	NEGATIVE	NEGATIVE	POSITIVE	NEGATIVE	NEGATIVE	POSITIVE	POSITIVE	NEGATIVE	POSITIVE	NEGATIVE
270	MANABÍ	SMALL	WARM	POSITIVE	NEGATIVE	NEGATIVE	POSITIVE	NEGATIVE	POSITIVE	NEGATIVE	POSITIVE	NEGATIVE	POSITIVE	NEGATIVE
272	MANABÍ	SMALL	WARM	POSITIVE	NEGATIVE	NEGATIVE	POSITIVE	NEGATIVE	POSITIVE	NEGATIVE	POSITIVE	NEGATIVE	POSITIVE	NEGATIVE
273	MANABÍ	MEDIUM	WARM	POSITIVE	NEGATIVE	NEGATIVE	POSITIVE	NEGATIVE	POSITIVE	NEGATIVE	POSITIVE	NEGATIVE	POSITIVE	POSITIVE
279	MANABÍ	MEDIUM	WARM	POSITIVE	NEGATIVE	NEGATIVE	POSITIVE	NEGATIVE	NEGATIVE	NEGATIVE	POSITIVE	NEGATIVE	POSITIVE	POSITIVE
283	MANABÍ	LARGE	WARM	POSITIVE	NEGATIVE	NEGATIVE	POSITIVE	NEGATIVE	POSITIVE	NEGATIVE	POSITIVE	NEGATIVE	POSITIVE	POSITIVE
284	MANABÍ	SMALL	WARM	POSITIVE	NEGATIVE	NEGATIVE	POSITIVE	NEGATIVE	POSITIVE	POSITIVE	POSITIVE	NEGATIVE	POSITIVE	NEGATIVE
287	MANABÍ	SMALL	WARM	POSITIVE	NEGATIVE	NEGATIVE	POSITIVE	NEGATIVE	NEGATIVE	NEGATIVE	POSITIVE	NEGATIVE	NEGATIVE	NEGATIVE
288	MANABÍ	MEDIUM	WARM	POSITIVE	NEGATIVE	NEGATIVE	POSITIVE	NEGATIVE	POSITIVE	NEGATIVE	POSITIVE	NEGATIVE	POSITIVE	NEGATIVE
289	MANABÍ	SMALL	WARM	POSITIVE	NEGATIVE	NEGATIVE	POSITIVE	NEGATIVE	POSITIVE	NEGATIVE	POSITIVE	NEGATIVE	POSITIVE	NEGATIVE
290	MANABÍ	LARGE	WARM	POSITIVE	NEGATIVE	NEGATIVE	POSITIVE	NEGATIVE	POSITIVE	NEGATIVE	POSITIVE	NEGATIVE	NEGATIVE	NEGATIVE
292	MANABÍ	MEDIUM	WARM	POSITIVE	NEGATIVE	NEGATIVE	POSITIVE	NEGATIVE	NEGATIVE	NEGATIVE	POSITIVE	NEGATIVE	NEGATIVE	NEGATIVE
293	MANABÍ	MEDIUM	WARM	POSITIVE	NEGATIVE	POSITIVE	POSITIVE	NEGATIVE	POSITIVE	POSITIVE	POSITIVE	NEGATIVE	NEGATIVE	NEGATIVE
295	MANABÍ	LARGE	WARM	POSITIVE	NEGATIVE	NEGATIVE	POSITIVE	NEGATIVE	NEGATIVE	NEGATIVE	POSITIVE	POSITIVE	POSITIVE	NEGATIVE
297	MANABÍ	MEDIUM	WARM	POSITIVE	NEGATIVE	NEGATIVE	POSITIVE	NEGATIVE	POSITIVE	NEGATIVE	POSITIVE	POSITIVE	POSITIVE	NEGATIVE
298	MANABÍ	LARGE	WARM	POSITIVE	NEGATIVE	NEGATIVE	POSITIVE	NEGATIVE	POSITIVE	NEGATIVE	POSITIVE	POSITIVE	POSITIVE	NEGATIVE
299	MANABÍ	LARGE	WARM	POSITIVE	NEGATIVE	NEGATIVE	POSITIVE	NEGATIVE	POSITIVE	NEGATIVE	POSITIVE	POSITIVE	POSITIVE	NEGATIVE
300	MANABÍ	MEDIUM	WARM	POSITIVE	NEGATIVE	NEGATIVE	POSITIVE	NEGATIVE	POSITIVE	NEGATIVE	POSITIVE	POSITIVE	POSITIVE	NEGATIVE
304	MANABÍ	LARGE	WARM	POSITIVE	NEGATIVE	NEGATIVE	POSITIVE	NEGATIVE	POSITIVE	POSITIVE	POSITIVE	NEGATIVE	POSITIVE	POSITIVE
307	MANABÍ	MEDIUM	WARM	POSITIVE	NEGATIVE	POSITIVE	POSITIVE	NEGATIVE	POSITIVE	POSITIVE	POSITIVE	NEGATIVE	POSITIVE	NEGATIVE
308	MANABÍ	SMALL	WARM	POSITIVE	NEGATIVE	POSITIVE	POSITIVE	NEGATIVE	NEGATIVE	POSITIVE	POSITIVE	NEGATIVE	POSITIVE	NEGATIVE
309	MANABÍ	LARGE	WARM	POSITIVE	NEGATIVE	NEGATIVE	POSITIVE	NEGATIVE	NEGATIVE	POSITIVE	POSITIVE	NEGATIVE	POSITIVE	NEGATIVE
312	MANABÍ	SMALL	WARM	POSITIVE	NEGATIVE	POSITIVE	POSITIVE	NEGATIVE	POSITIVE	POSITIVE	POSITIVE	NEGATIVE	POSITIVE	NEGATIVE
315	MANABÍ	MEDIUM	WARM	POSITIVE	NEGATIVE	NEGATIVE	POSITIVE	NEGATIVE	NEGATIVE	POSITIVE	POSITIVE	NEGATIVE	POSITIVE	NEGATIVE
317	MANABÍ	SMALL	WARM	POSITIVE	NEGATIVE	NEGATIVE	POSITIVE	NEGATIVE	POSITIVE	POSITIVE	POSITIVE	NEGATIVE	POSITIVE	NEGATIVE
333	PICHINCHA	LARGE	WARM	POSITIVE	NEGATIVE	NEGATIVE	POSITIVE	NEGATIVE	POSITIVE	POSITIVE	POSITIVE	NEGATIVE	NEGATIVE	NEGATIVE
334	PICHINCHA	MEDIUM	WARM	POSITIVE	NEGATIVE	NEGATIVE	POSITIVE	NEGATIVE	POSITIVE	NEGATIVE	POSITIVE	NEGATIVE	NEGATIVE	NEGATIVE
335	PICHINCHA	SMALL	WARM	POSITIVE	NEGATIVE	NEGATIVE	POSITIVE	NEGATIVE	POSITIVE	POSITIVE	NEGATIVE	NEGATIVE	NEGATIVE	NEGATIVE
338	PICHINCHA	SMALL	WARM	POSITIVE	NEGATIVE	NEGATIVE	POSITIVE	NEGATIVE	POSITIVE	NEGATIVE	POSITIVE	NEGATIVE	POSITIVE	NEGATIVE
340	PICHINCHA	MEDIUM	WARM	POSITIVE	NEGATIVE	NEGATIVE	POSITIVE	NEGATIVE	POSITIVE	NEGATIVE	POSITIVE	NEGATIVE	POSITIVE	NEGATIVE
342	PICHINCHA	SMALL	WARM	POSITIVE	NEGATIVE	NEGATIVE	POSITIVE	NEGATIVE	POSITIVE	NEGATIVE	NEGATIVE	NEGATIVE	POSITIVE	NEGATIVE
344	PICHINCHA	SMALL	WARM	POSITIVE	POSITIVE	NEGATIVE	POSITIVE	NEGATIVE	POSITIVE	POSITIVE	NEGATIVE	NEGATIVE	POSITIVE	NEGATIVE
347	PICHINCHA	LARGE	WARM	POSITIVE	POSITIVE	NEGATIVE	POSITIVE	NEGATIVE	POSITIVE	POSITIVE	POSITIVE	NEGATIVE	POSITIVE	NEGATIVE
348	PICHINCHA	SMALL	WARM	POSITIVE	NEGATIVE	NEGATIVE	POSITIVE	NEGATIVE	POSITIVE	POSITIVE	POSITIVE	NEGATIVE	POSITIVE	NEGATIVE
351	PICHINCHA	LARGE	WARM	POSITIVE	NEGATIVE	NEGATIVE	POSITIVE	NEGATIVE	NEGATIVE	NEGATIVE	NEGATIVE	NEGATIVE	POSITIVE	NEGATIVE
352	PICHINCHA	SMALL	WARM	POSITIVE	POSITIVE	POSITIVE	POSITIVE	NEGATIVE	POSITIVE	NEGATIVE	POSITIVE	NEGATIVE	NEGATIVE	NEGATIVE
353	PICHINCHA	SMALL	WARM	POSITIVE	POSITIVE	NEGATIVE	POSITIVE	NEGATIVE	NEGATIVE	NEGATIVE	POSITIVE	NEGATIVE	NEGATIVE	NEGATIVE
354	PICHINCHA	MEDIUM	WARM	POSITIVE	POSITIVE	NEGATIVE	NEGATIVE	NEGATIVE	NEGATIVE	NEGATIVE	NEGATIVE	NEGATIVE	NEGATIVE	NEGATIVE
355	PICHINCHA	MEDIUM	WARM	POSITIVE	POSITIVE	NEGATIVE	NEGATIVE	NEGATIVE	NEGATIVE	NEGATIVE	POSITIVE	NEGATIVE	POSITIVE	NEGATIVE
356	PICHINCHA	SMALL	WARM	POSITIVE	POSITIVE	NEGATIVE	NEGATIVE	NEGATIVE	POSITIVE	NEGATIVE	POSITIVE	NEGATIVE	POSITIVE	NEGATIVE
358	PICHINCHA	SMALL	WARM	POSITIVE	POSITIVE	NEGATIVE	NEGATIVE	NEGATIVE	NEGATIVE	NEGATIVE	POSITIVE	NEGATIVE	NEGATIVE	NEGATIVE
359	PICHINCHA	LARGE	WARM	POSITIVE	NEGATIVE	NEGATIVE	POSITIVE	NEGATIVE	POSITIVE	NEGATIVE	POSITIVE	NEGATIVE	NEGATIVE	NEGATIVE
361	PICHINCHA	LARGE	WARM	POSITIVE	POSITIVE	NEGATIVE	POSITIVE	NEGATIVE	NEGATIVE	NEGATIVE	POSITIVE	NEGATIVE	NEGATIVE	NEGATIVE
363	PICHINCHA	SMALL	WARM	POSITIVE	POSITIVE	POSITIVE	POSITIVE	NEGATIVE	NEGATIVE	NEGATIVE	POSITIVE	NEGATIVE	POSITIVE	NEGATIVE
364	PICHINCHA	MEDIUM	WARM	POSITIVE	POSITIVE	NEGATIVE	POSITIVE	NEGATIVE	NEGATIVE	NEGATIVE	POSITIVE	NEGATIVE	POSITIVE	NEGATIVE
365	PICHINCHA	SMALL	WARM	POSITIVE	NEGATIVE	NEGATIVE	POSITIVE	NEGATIVE	NEGATIVE	NEGATIVE	POSITIVE	NEGATIVE	POSITIVE	NEGATIVE
368	PICHINCHA	SMALL	WARM	POSITIVE	POSITIVE	NEGATIVE	POSITIVE	NEGATIVE	NEGATIVE	NEGATIVE	POSITIVE	NEGATIVE	NEGATIVE	NEGATIVE
369	PICHINCHA	LARGE	WARM	POSITIVE	POSITIVE	NEGATIVE	POSITIVE	NEGATIVE	NEGATIVE	NEGATIVE	NEGATIVE	NEGATIVE	NEGATIVE	NEGATIVE
370	PICHINCHA	SMALL	WARM	POSITIVE	POSITIVE	NEGATIVE	POSITIVE	NEGATIVE	NEGATIVE	NEGATIVE	NEGATIVE	NEGATIVE	POSITIVE	NEGATIVE
371	PICHINCHA	MEDIUM	WARM	POSITIVE	NEGATIVE	NEGATIVE	POSITIVE	NEGATIVE	NEGATIVE	NEGATIVE	POSITIVE	NEGATIVE	POSITIVE	NEGATIVE
375	PICHINCHA	MEDIUM	WARM	POSITIVE	POSITIVE	NEGATIVE	NEGATIVE	NEGATIVE	NEGATIVE	NEGATIVE	POSITIVE	NEGATIVE	NEGATIVE	NEGATIVE
376	PICHINCHA	MEDIUM	WARM	POSITIVE	POSITIVE	NEGATIVE	POSITIVE	NEGATIVE	NEGATIVE	NEGATIVE	POSITIVE	NEGATIVE	POSITIVE	NEGATIVE
378	PICHINCHA	MEDIUM	WARM	POSITIVE	POSITIVE	NEGATIVE	POSITIVE	NEGATIVE	NEGATIVE	NEGATIVE	POSITIVE	NEGATIVE	POSITIVE	NEGATIVE
381	PICHINCHA	LARGE	WARM	POSITIVE	NEGATIVE	NEGATIVE	POSITIVE	NEGATIVE	NEGATIVE	NEGATIVE	POSITIVE	NEGATIVE	POSITIVE	NEGATIVE
382	PICHINCHA	SMALL	WARM	POSITIVE	POSITIVE	NEGATIVE	POSITIVE	NEGATIVE	NEGATIVE	NEGATIVE	POSITIVE	NEGATIVE	NEGATIVE	NEGATIVE
384	PICHINCHA	LARGE	WARM	POSITIVE	POSITIVE	NEGATIVE	POSITIVE	NEGATIVE	NEGATIVE	NEGATIVE	POSITIVE	NEGATIVE	POSITIVE	NEGATIVE
387	PICHINCHA	SMALL	WARM	POSITIVE	NEGATIVE	NEGATIVE	POSITIVE	NEGATIVE	NEGATIVE	NEGATIVE	POSITIVE	NEGATIVE	POSITIVE	NEGATIVE
389	PICHINCHA	SMALL	WARM	POSITIVE	NEGATIVE	NEGATIVE	POSITIVE	NEGATIVE	NEGATIVE	NEGATIVE	POSITIVE	NEGATIVE	POSITIVE	NEGATIVE

390	PICHINCHA	LARGE	WARM	POSITIVE	POSITIVE	NEGATIVE	POSITIVE	NEGATIVE	NEGATIVE	NEGATIVE	POSITIVE	NEGATIVE	POSITIVE	NEGATIVE
391	PICHINCHA	SMALL	WARM	POSITIVE	POSITIVE	POSITIVE	POSITIVE	NEGATIVE	NEGATIVE	NEGATIVE	NEGATIVE	NEGATIVE	POSITIVE	NEGATIVE
394	PICHINCHA	SMALL	WARM	POSITIVE	POSITIVE	POSITIVE	POSITIVE	NEGATIVE	NEGATIVE	NEGATIVE	POSITIVE	NEGATIVE	POSITIVE	NEGATIVE
395	PICHINCHA	MEDIUM	WARM	POSITIVE	POSITIVE	NEGATIVE	POSITIVE	NEGATIVE	NEGATIVE	NEGATIVE	NEGATIVE	POSITIVE	POSITIVE	NEGATIVE
396	MANABÍ	MEDIUM	WARM	POSITIVE	POSITIVE	POSITIVE	POSITIVE	NEGATIVE	NEGATIVE	NEGATIVE	POSITIVE	NEGATIVE	POSITIVE	NEGATIVE
398	MANABÍ	MEDIUM	WARM	POSITIVE	POSITIVE	NEGATIVE	NEGATIVE	NEGATIVE	NEGATIVE	NEGATIVE	NEGATIVE	NEGATIVE	NEGATIVE	NEGATIVE
399	MANABÍ	SMALL	WARM	POSITIVE	POSITIVE	NEGATIVE	POSITIVE	NEGATIVE	NEGATIVE	NEGATIVE	POSITIVE	NEGATIVE	NEGATIVE	NEGATIVE
400	MANABÍ	SMALL	WARM	POSITIVE	POSITIVE	NEGATIVE	POSITIVE	POSITIVE	NEGATIVE	NEGATIVE	POSITIVE	NEGATIVE	NEGATIVE	NEGATIVE
401	MANABÍ	LARGE	WARM	POSITIVE	POSITIVE	NEGATIVE	NEGATIVE	NEGATIVE	NEGATIVE	NEGATIVE	NEGATIVE	NEGATIVE	NEGATIVE	NEGATIVE
403	MANABÍ	LARGE	WARM	POSITIVE	POSITIVE	NEGATIVE	POSITIVE	NEGATIVE	POSITIVE	NEGATIVE	POSITIVE	NEGATIVE	NEGATIVE	NEGATIVE
404	MANABÍ	SMALL	WARM	POSITIVE	POSITIVE	NEGATIVE	POSITIVE	NEGATIVE	POSITIVE	NEGATIVE	POSITIVE	NEGATIVE	POSITIVE	NEGATIVE
405	MANABÍ	MEDIUM	WARM	POSITIVE	POSITIVE	NEGATIVE	POSITIVE	NEGATIVE	NEGATIVE	NEGATIVE	POSITIVE	NEGATIVE	POSITIVE	NEGATIVE
408	MANABÍ	LARGE	WARM	POSITIVE	POSITIVE	NEGATIVE	POSITIVE	NEGATIVE	POSITIVE	NEGATIVE	POSITIVE	NEGATIVE	NEGATIVE	NEGATIVE
409	MANABÍ	SMALL	WARM	POSITIVE	POSITIVE	NEGATIVE	POSITIVE	NEGATIVE	POSITIVE	NEGATIVE	NEGATIVE	NEGATIVE	NEGATIVE	NEGATIVE
410	MANABÍ	LARGE	WARM	POSITIVE	POSITIVE	NEGATIVE	POSITIVE	NEGATIVE	NEGATIVE	NEGATIVE	NEGATIVE	NEGATIVE	NEGATIVE	NEGATIVE
411	MANABÍ	LARGE	WARM	POSITIVE	POSITIVE	NEGATIVE	POSITIVE	NEGATIVE	NEGATIVE	NEGATIVE	POSITIVE	NEGATIVE	POSITIVE	NEGATIVE
412	MANABÍ	LARGE	WARM	POSITIVE	POSITIVE	NEGATIVE	POSITIVE	NEGATIVE	NEGATIVE	NEGATIVE	POSITIVE	NEGATIVE	POSITIVE	NEGATIVE
413	MANABÍ	SMALL	WARM	POSITIVE	POSITIVE	NEGATIVE	POSITIVE	NEGATIVE	NEGATIVE	NEGATIVE	POSITIVE	NEGATIVE	POSITIVE	NEGATIVE
414	MANABÍ	SMALL	WARM	POSITIVE	POSITIVE	NEGATIVE	NEGATIVE	NEGATIVE	NEGATIVE	NEGATIVE	POSITIVE	NEGATIVE	NEGATIVE	NEGATIVE
415	MANABÍ	MEDIUM	WARM	POSITIVE	POSITIVE	NEGATIVE	POSITIVE	NEGATIVE	NEGATIVE	NEGATIVE	POSITIVE	NEGATIVE	NEGATIVE	NEGATIVE
418	MANABÍ	SMALL	WARM	POSITIVE	POSITIVE	NEGATIVE	POSITIVE	NEGATIVE	NEGATIVE	NEGATIVE	POSITIVE	NEGATIVE	NEGATIVE	NEGATIVE
420	MANABÍ	LARGE	WARM	POSITIVE	POSITIVE	NEGATIVE	POSITIVE	NEGATIVE	NEGATIVE	NEGATIVE	POSITIVE	NEGATIVE	POSITIVE	NEGATIVE
421	MANABÍ	MEDIUM	WARM	POSITIVE	POSITIVE	NEGATIVE	POSITIVE	NEGATIVE	NEGATIVE	NEGATIVE	POSITIVE	POSITIVE	POSITIVE	NEGATIVE
422	MANABÍ	MEDIUM	WARM	POSITIVE	POSITIVE	NEGATIVE	POSITIVE	NEGATIVE	POSITIVE	NEGATIVE	POSITIVE	NEGATIVE	NEGATIVE	NEGATIVE
424	MANABÍ	LARGE	WARM	POSITIVE	POSITIVE	NEGATIVE	POSITIVE	NEGATIVE	POSITIVE	NEGATIVE	POSITIVE	NEGATIVE	NEGATIVE	NEGATIVE
425	MANABÍ	SMALL	WARM	POSITIVE	POSITIVE	NEGATIVE	POSITIVE	NEGATIVE	POSITIVE	NEGATIVE	POSITIVE	NEGATIVE	NEGATIVE	NEGATIVE
442	MANABÍ	MEDIUM	WARM	POSITIVE	POSITIVE	NEGATIVE	POSITIVE	NEGATIVE	NEGATIVE	NEGATIVE	POSITIVE	NEGATIVE	POSITIVE	NEGATIVE
444	MANABÍ	SMALL	WARM	POSITIVE	POSITIVE	POSITIVE	POSITIVE	NEGATIVE	NEGATIVE	NEGATIVE	POSITIVE	NEGATIVE	POSITIVE	NEGATIVE
445	MANABÍ	LARGE	WARM	POSITIVE	POSITIVE	NEGATIVE	POSITIVE	NEGATIVE	POSITIVE	NEGATIVE	POSITIVE	NEGATIVE	POSITIVE	NEGATIVE
449	MANABÍ	SMALL	WARM	POSITIVE	POSITIVE	NEGATIVE	POSITIVE	NEGATIVE	POSITIVE	NEGATIVE	POSITIVE	NEGATIVE	POSITIVE	NEGATIVE
450	MANABÍ	SMALL	WARM	POSITIVE	POSITIVE	NEGATIVE	POSITIVE	NEGATIVE	NEGATIVE	NEGATIVE	POSITIVE	NEGATIVE	POSITIVE	NEGATIVE
452	PICHINCHA	SMALL	WARM	POSITIVE	POSITIVE	NEGATIVE	POSITIVE	NEGATIVE	NEGATIVE	NEGATIVE	POSITIVE	NEGATIVE	POSITIVE	NEGATIVE
453	PICHINCHA	SMALL	WARM	POSITIVE	POSITIVE	NEGATIVE	POSITIVE	NEGATIVE	POSITIVE	NEGATIVE	POSITIVE	NEGATIVE	NEGATIVE	NEGATIVE
454	PICHINCHA	MEDIUM	WARM	POSITIVE	POSITIVE	NEGATIVE	POSITIVE	NEGATIVE	NEGATIVE	NEGATIVE	POSITIVE	NEGATIVE	POSITIVE	NEGATIVE
455	PICHINCHA	SMALL	WARM	POSITIVE	NEGATIVE	NEGATIVE	POSITIVE	NEGATIVE	NEGATIVE	NEGATIVE	POSITIVE	NEGATIVE	POSITIVE	NEGATIVE
460	MANABÍ	SMALL	WARM	POSITIVE	NEGATIVE	NEGATIVE	POSITIVE	NEGATIVE	NEGATIVE	NEGATIVE	POSITIVE	NEGATIVE	POSITIVE	NEGATIVE
461	MANABÍ	SMALL	WARM	POSITIVE	NEGATIVE	NEGATIVE	POSITIVE	NEGATIVE	NEGATIVE	NEGATIVE	POSITIVE	NEGATIVE	POSITIVE	NEGATIVE
462	MANABÍ	LARGE	WARM	POSITIVE	NEGATIVE	NEGATIVE	POSITIVE	NEGATIVE	NEGATIVE	NEGATIVE	POSITIVE	NEGATIVE	POSITIVE	NEGATIVE
463	MANABÍ	SMALL	WARM	POSITIVE	NEGATIVE	NEGATIVE	POSITIVE	POSITIVE	NEGATIVE	NEGATIVE	POSITIVE	NEGATIVE	POSITIVE	NEGATIVE
464	MANABÍ	LARGE	WARM	POSITIVE	NEGATIVE	NEGATIVE	POSITIVE	NEGATIVE	NEGATIVE	NEGATIVE	POSITIVE	NEGATIVE	POSITIVE	NEGATIVE
466	MANABÍ	MEDIUM	WARM	POSITIVE	NEGATIVE	NEGATIVE	POSITIVE	NEGATIVE	NEGATIVE	NEGATIVE	NEGATIVE	NEGATIVE	POSITIVE	NEGATIVE
468	MANABÍ	LARGE	WARM	POSITIVE	NEGATIVE	NEGATIVE	POSITIVE	NEGATIVE	NEGATIVE	NEGATIVE	POSITIVE	NEGATIVE	NEGATIVE	NEGATIVE
469	MANABÍ	LARGE	WARM	POSITIVE	NEGATIVE	NEGATIVE	POSITIVE	NEGATIVE	NEGATIVE	NEGATIVE	POSITIVE	NEGATIVE	NEGATIVE	NEGATIVE
471	MANABÍ	LARGE	WARM	POSITIVE	NEGATIVE	NEGATIVE	POSITIVE	NEGATIVE	NEGATIVE	NEGATIVE	POSITIVE	NEGATIVE	POSITIVE	NEGATIVE
473	MANABÍ	LARGE	WARM	POSITIVE	NEGATIVE	NEGATIVE	POSITIVE	NEGATIVE	NEGATIVE	NEGATIVE	POSITIVE	NEGATIVE	POSITIVE	NEGATIVE
474	MANABÍ	SMALL	WARM	POSITIVE	NEGATIVE	NEGATIVE	POSITIVE	NEGATIVE	NEGATIVE	NEGATIVE	POSITIVE	NEGATIVE	POSITIVE	NEGATIVE
475	MANABÍ	SMALL	WARM	POSITIVE	NEGATIVE	NEGATIVE	POSITIVE	NEGATIVE	NEGATIVE	NEGATIVE	POSITIVE	NEGATIVE	POSITIVE	NEGATIVE
476	MANABÍ	MEDIUM	WARM	POSITIVE	NEGATIVE	NEGATIVE	POSITIVE	NEGATIVE	NEGATIVE	NEGATIVE	POSITIVE	NEGATIVE	POSITIVE	NEGATIVE
477	MANABÍ	SMALL	WARM	POSITIVE	NEGATIVE	NEGATIVE	POSITIVE	NEGATIVE	NEGATIVE	NEGATIVE	POSITIVE	NEGATIVE	POSITIVE	NEGATIVE
479	MANABÍ	SMALL	WARM	POSITIVE	NEGATIVE	NEGATIVE	POSITIVE	NEGATIVE	NEGATIVE	NEGATIVE	POSITIVE	NEGATIVE	NEGATIVE	NEGATIVE
480	MANABÍ	SMALL	WARM	POSITIVE	NEGATIVE	NEGATIVE	POSITIVE	NEGATIVE	NEGATIVE	NEGATIVE	POSITIVE	NEGATIVE	NEGATIVE	NEGATIVE
481	MANABÍ	LARGE	WARM	POSITIVE	NEGATIVE	NEGATIVE	POSITIVE	NEGATIVE	NEGATIVE	NEGATIVE	NEGATIVE	NEGATIVE	NEGATIVE	NEGATIVE
483	MANABÍ	LARGE	WARM	POSITIVE	NEGATIVE	POSITIVE	POSITIVE	NEGATIVE	NEGATIVE	NEGATIVE	POSITIVE	NEGATIVE	NEGATIVE	NEGATIVE
485	PICHINCHA	MEDIUM	WARM	POSITIVE	NEGATIVE	POSITIVE	POSITIVE	POSITIVE	NEGATIVE	NEGATIVE	POSITIVE	NEGATIVE	NEGATIVE	NEGATIVE
486	PICHINCHA	SMALL	WARM	POSITIVE	NEGATIVE	NEGATIVE	POSITIVE	NEGATIVE	POSITIVE	NEGATIVE	POSITIVE	NEGATIVE	NEGATIVE	NEGATIVE
487	PICHINCHA	LARGE	WARM	POSITIVE	NEGATIVE	NEGATIVE	POSITIVE	NEGATIVE	POSITIVE	NEGATIVE	POSITIVE	POSITIVE	POSITIVE	NEGATIVE
490	PICHINCHA	MEDIUM	WARM	POSITIVE	NEGATIVE	NEGATIVE	POSITIVE	NEGATIVE	NEGATIVE	NEGATIVE	POSITIVE	POSITIVE	POSITIVE	NEGATIVE
491	PICHINCHA	MEDIUM	WARM	POSITIVE	NEGATIVE	NEGATIVE	POSITIVE	NEGATIVE	NEGATIVE	NEGATIVE	POSITIVE	NEGATIVE	NEGATIVE	NEGATIVE
494	PICHINCHA	LARGE	WARM	POSITIVE	NEGATIVE	NEGATIVE	POSITIVE	NEGATIVE	NEGATIVE	NEGATIVE	POSITIVE	NEGATIVE	NEGATIVE	NEGATIVE
495	PICHINCHA	LARGE	WARM	POSITIVE	NEGATIVE	POSITIVE	POSITIVE	NEGATIVE	NEGATIVE	NEGATIVE	POSITIVE	NEGATIVE	NEGATIVE	NEGATIVE
496	PICHINCHA	SMALL	WARM	POSITIVE	NEGATIVE	NEGATIVE	POSITIVE	NEGATIVE	NEGATIVE	NEGATIVE	POSITIVE	NEGATIVE	NEGATIVE	NEGATIVE
497	PICHINCHA	MEDIUM	WARM	POSITIVE	NEGATIVE	POSITIVE	POSITIVE	NEGATIVE	NEGATIVE	NEGATIVE	NEGATIVE	NEGATIVE	NEGATIVE	NEGATIVE
498	PICHINCHA	SMALL	WARM	POSITIVE	NEGATIVE	POSITIVE	POSITIVE	NEGATIVE	NEGATIVE	NEGATIVE	POSITIVE	NEGATIVE	POSITIVE	NEGATIVE
500	PICHINCHA	SMALL	WARM	POSITIVE	NEGATIVE	NEGATIVE	POSITIVE	NEGATIVE	NEGATIVE	NEGATIVE	POSITIVE	NEGATIVE	NEGATIVE	NEGATIVE
501	PICHINCHA	LARGE	WARM	POSITIVE	NEGATIVE	NEGATIVE	POSITIVE	NEGATIVE	NEGATIVE	NEGATIVE	POSITIVE	NEGATIVE	NEGATIVE	NEGATIVE
504	PICHINCHA	SMALL	WARM	POSITIVE	NEGATIVE	NEGATIVE	POSITIVE	NEGATIVE	POSITIVE	NEGATIVE	NEGATIVE	NEGATIVE	NEGATIVE	NEGATIVE
505	PICHINCHA	SMALL	WARM	POSITIVE	NEGATIVE	NEGATIVE	POSITIVE	NEGATIVE	POSITIVE	NEGATIVE	POSITIVE	NEGATIVE	POSITIVE	NEGATIVE
507	PICHINCHA	SMALL	WARM	POSITIVE	NEGATIVE	NEGATIVE	POSITIVE	NEGATIVE	NEGATIVE	NEGATIVE	POSITIVE	NEGATIVE	POSITIVE	NEGATIVE
508	PICHINCHA	MEDIUM	WARM	POSITIVE	NEGATIVE	NEGATIVE	POSITIVE	NEGATIVE	NEGATIVE	NEGATIVE	POSITIVE	NEGATIVE	NEGATIVE	NEGATIVE
509	PICHINCHA	SMALL	WARM	POSITIVE	NEGATIVE	NEGATIVE	POSITIVE	NEGATIVE	NEGATIVE	NEGATIVE	POSITIVE	NEGATIVE	POSITIVE	NEGATIVE
510	PICHINCHA	LARGE	WARM	POSITIVE	NEGATIVE	NEGATIVE	POSITIVE	NEGATIVE	NEGATIVE	NEGATIVE	POSITIVE	NEGATIVE	POSITIVE	NEGATIVE
511	PICHINCHA	MEDIUM	WARM	POSITIVE	NEGATIVE	NEGATIVE	POSITIVE	NEGATIVE	NEGATIVE	NEGATIVE	POSITIVE	NEGATIVE	NEGATIVE	NEGATIVE
513	PICHINCHA	LARGE	WARM	POSITIVE	NEGATIVE	NEGATIVE	POSITIVE	NEGATIVE	NEGATIVE	NEGATIVE	POSITIVE	NEGATIVE	NEGATIVE	NEGATIVE
514	PICHINCHA	SMALL	WARM	POSITIVE	NEGATIVE	POSITIVE	POSITIVE	NEGATIVE	POSITIVE	NEGATIVE	POSITIVE	NEGATIVE	POSITIVE	POSITIVE

515	PICHINCHA	SMALL	WARM	POSITIVE	NEGATIVE	NEGATIVE	NEGATIVE	NEGATIVE	NEGATIVE	NEGATIVE	POSITIVE	NEGATIVE	NEGATIVE	NEGATIVE
516	MANABÍ	MEDIUM	WARM	POSITIVE	NEGATIVE	NEGATIVE	POSITIVE	NEGATIVE	POSITIVE	NEGATIVE	POSITIVE	NEGATIVE	POSITIVE	POSITIVE
517	MANABÍ	MEDIUM	WARM	POSITIVE	NEGATIVE	NEGATIVE	POSITIVE	NEGATIVE	POSITIVE	NEGATIVE	NEGATIVE	NEGATIVE	POSITIVE	POSITIVE
518	MANABÍ	LARGE	WARM	POSITIVE	NEGATIVE	POSITIVE	POSITIVE	NEGATIVE	POSITIVE	NEGATIVE	POSITIVE	NEGATIVE	POSITIVE	POSITIVE
519	MANABÍ	SMALL	WARM	POSITIVE	NEGATIVE	POSITIVE	POSITIVE	NEGATIVE	NEGATIVE	NEGATIVE	POSITIVE	POSITIVE	POSITIVE	NEGATIVE
522	MANABÍ	LARGE	WARM	POSITIVE	NEGATIVE	POSITIVE	POSITIVE	NEGATIVE	POSITIVE	NEGATIVE	POSITIVE	POSITIVE	POSITIVE	NEGATIVE
526	MANABÍ	MEDIUM	WARM	POSITIVE	NEGATIVE	NEGATIVE	POSITIVE	NEGATIVE	NEGATIVE	NEGATIVE	POSITIVE	NEGATIVE	NEGATIVE	NEGATIVE
527	MANABÍ	SMALL	WARM	POSITIVE	NEGATIVE	NEGATIVE	POSITIVE	NEGATIVE	NEGATIVE	NEGATIVE	POSITIVE	NEGATIVE	NEGATIVE	POSITIVE
528	MANABÍ	MEDIUM	WARM	POSITIVE	NEGATIVE	POSITIVE	POSITIVE	NEGATIVE	POSITIVE	NEGATIVE	POSITIVE	NEGATIVE	POSITIVE	POSITIVE
529	MANABÍ	SMALL	WARM	POSITIVE	NEGATIVE	NEGATIVE	POSITIVE	NEGATIVE	NEGATIVE	NEGATIVE	POSITIVE	NEGATIVE	NEGATIVE	NEGATIVE
534	MANABÍ	LARGE	WARM	POSITIVE	NEGATIVE	NEGATIVE	POSITIVE	NEGATIVE	NEGATIVE	NEGATIVE	POSITIVE	NEGATIVE	NEGATIVE	NEGATIVE
535	MANABÍ	SMALL	WARM	POSITIVE	NEGATIVE	NEGATIVE	POSITIVE	NEGATIVE	NEGATIVE	NEGATIVE	POSITIVE	NEGATIVE	POSITIVE	POSITIVE
536	MANABÍ	SMALL	WARM	POSITIVE	NEGATIVE	NEGATIVE	POSITIVE	NEGATIVE	NEGATIVE	NEGATIVE	POSITIVE	POSITIVE	POSITIVE	POSITIVE
539	MANABÍ	SMALL	WARM	POSITIVE	NEGATIVE	NEGATIVE	POSITIVE	NEGATIVE	NEGATIVE	NEGATIVE	POSITIVE	NEGATIVE	POSITIVE	POSITIVE
540	MANABÍ	MEDIUM	WARM	POSITIVE	NEGATIVE	POSITIVE	POSITIVE	NEGATIVE	NEGATIVE	NEGATIVE	POSITIVE	NEGATIVE	NEGATIVE	NEGATIVE
544	MANABÍ	LARGE	WARM	POSITIVE	NEGATIVE	NEGATIVE	POSITIVE	NEGATIVE	NEGATIVE	NEGATIVE	POSITIVE	NEGATIVE	POSITIVE	NEGATIVE
545	MANABÍ	MEDIUM	WARM	POSITIVE	NEGATIVE	NEGATIVE	POSITIVE	NEGATIVE	NEGATIVE	NEGATIVE	POSITIVE	NEGATIVE	POSITIVE	NEGATIVE
547	MANABÍ	MEDIUM	WARM	POSITIVE	NEGATIVE	NEGATIVE	POSITIVE	NEGATIVE	NEGATIVE	NEGATIVE	NEGATIVE	NEGATIVE	POSITIVE	NEGATIVE
549	MANABÍ	LARGE	WARM	POSITIVE	NEGATIVE	NEGATIVE	POSITIVE	NEGATIVE	NEGATIVE	NEGATIVE	NEGATIVE	NEGATIVE	POSITIVE	NEGATIVE
553	MANABÍ	MEDIUM	WARM	POSITIVE	NEGATIVE	NEGATIVE	POSITIVE	POSITIVE	NEGATIVE	NEGATIVE	NEGATIVE	NEGATIVE	POSITIVE	POSITIVE
556	MANABÍ	MEDIUM	WARM	POSITIVE	NEGATIVE	NEGATIVE	POSITIVE	NEGATIVE	NEGATIVE	NEGATIVE	NEGATIVE	NEGATIVE	POSITIVE	NEGATIVE
557	MANABÍ	MEDIUM	WARM	POSITIVE	NEGATIVE	NEGATIVE	POSITIVE	NEGATIVE	NEGATIVE	NEGATIVE	NEGATIVE	NEGATIVE	NEGATIVE	NEGATIVE
558	MANABÍ	LARGE	WARM	POSITIVE	NEGATIVE	NEGATIVE	POSITIVE	NEGATIVE	NEGATIVE	NEGATIVE	POSITIVE	NEGATIVE	POSITIVE	NEGATIVE
566	MANABÍ	SMALL	WARM	POSITIVE	NEGATIVE	NEGATIVE	POSITIVE	NEGATIVE	NEGATIVE	NEGATIVE	POSITIVE	NEGATIVE	NEGATIVE	NEGATIVE
567	MANABÍ	MEDIUM	WARM	POSITIVE	NEGATIVE	NEGATIVE	POSITIVE	NEGATIVE	NEGATIVE	NEGATIVE	POSITIVE	POSITIVE	POSITIVE	NEGATIVE
569	MANABÍ	SMALL	WARM	POSITIVE	NEGATIVE	POSITIVE	POSITIVE	NEGATIVE	NEGATIVE	NEGATIVE	POSITIVE	POSITIVE	POSITIVE	POSITIVE
570	MANABÍ	MEDIUM	WARM	POSITIVE	NEGATIVE	NEGATIVE	POSITIVE	NEGATIVE	NEGATIVE	NEGATIVE	NEGATIVE	NEGATIVE	POSITIVE	NEGATIVE
572	MANABÍ	LARGE	WARM	POSITIVE	NEGATIVE	POSITIVE	POSITIVE	NEGATIVE	NEGATIVE	NEGATIVE	NEGATIVE	NEGATIVE	POSITIVE	NEGATIVE
578	PICHINCHA	LARGE	WARM	POSITIVE	NEGATIVE	NEGATIVE	POSITIVE	NEGATIVE	NEGATIVE	NEGATIVE	POSITIVE	NEGATIVE	POSITIVE	NEGATIVE
580	PICHINCHA	MEDIUM	WARM	POSITIVE	NEGATIVE	NEGATIVE	POSITIVE	NEGATIVE	NEGATIVE	NEGATIVE	NEGATIVE	NEGATIVE	POSITIVE	NEGATIVE
581	PICHINCHA	MEDIUM	WARM	POSITIVE	NEGATIVE	NEGATIVE	POSITIVE	NEGATIVE	NEGATIVE	NEGATIVE	NEGATIVE	NEGATIVE	POSITIVE	NEGATIVE
585	PICHINCHA	LARGE	WARM	POSITIVE	NEGATIVE	NEGATIVE	POSITIVE	NEGATIVE	NEGATIVE	NEGATIVE	NEGATIVE	NEGATIVE	POSITIVE	NEGATIVE
590	PICHINCHA	LARGE	WARM	POSITIVE	NEGATIVE	NEGATIVE	POSITIVE	NEGATIVE	NEGATIVE	NEGATIVE	NEGATIVE	NEGATIVE	POSITIVE	NEGATIVE
591	PICHINCHA	SMALL	WARM	POSITIVE	NEGATIVE	NEGATIVE	POSITIVE	POSITIVE	NEGATIVE	NEGATIVE	NEGATIVE	NEGATIVE	POSITIVE	NEGATIVE
592	PICHINCHA	MEDIUM	WARM	POSITIVE	NEGATIVE	NEGATIVE	POSITIVE	NEGATIVE	NEGATIVE	NEGATIVE	NEGATIVE	NEGATIVE	POSITIVE	NEGATIVE
593	PICHINCHA	SMALL	WARM	POSITIVE	NEGATIVE	NEGATIVE	POSITIVE	NEGATIVE	NEGATIVE	NEGATIVE	NEGATIVE	NEGATIVE	POSITIVE	NEGATIVE
594	PICHINCHA	LARGE	WARM	POSITIVE	NEGATIVE	NEGATIVE	POSITIVE	NEGATIVE	NEGATIVE	NEGATIVE	NEGATIVE	NEGATIVE	POSITIVE	NEGATIVE
595	PICHINCHA	MEDIUM	WARM	POSITIVE	NEGATIVE	NEGATIVE	POSITIVE	NEGATIVE	NEGATIVE	NEGATIVE	NEGATIVE	NEGATIVE	POSITIVE	NEGATIVE
602	PICHINCHA	SMALL	WARM	POSITIVE	NEGATIVE	NEGATIVE	POSITIVE	NEGATIVE	NEGATIVE	NEGATIVE	NEGATIVE	NEGATIVE	POSITIVE	NEGATIVE
605	PICHINCHA	MEDIUM	WARM	POSITIVE	NEGATIVE	NEGATIVE	POSITIVE	NEGATIVE	NEGATIVE	NEGATIVE	NEGATIVE	NEGATIVE	POSITIVE	NEGATIVE
606	PICHINCHA	MEDIUM	WARM	POSITIVE	NEGATIVE	NEGATIVE	POSITIVE	POSITIVE	NEGATIVE	NEGATIVE	NEGATIVE	NEGATIVE	POSITIVE	NEGATIVE
608	PICHINCHA	MEDIUM	WARM	POSITIVE	NEGATIVE	NEGATIVE	POSITIVE	NEGATIVE	NEGATIVE	NEGATIVE	NEGATIVE	NEGATIVE	POSITIVE	NEGATIVE
612	PICHINCHA	SMALL	WARM	POSITIVE	NEGATIVE	NEGATIVE	POSITIVE	NEGATIVE	NEGATIVE	NEGATIVE	NEGATIVE	NEGATIVE	POSITIVE	NEGATIVE
616	PICHINCHA	LARGE	WARM	POSITIVE	NEGATIVE	NEGATIVE	POSITIVE	NEGATIVE	NEGATIVE	NEGATIVE	NEGATIVE	NEGATIVE	POSITIVE	NEGATIVE
619	PICHINCHA	SMALL	WARM	POSITIVE	NEGATIVE	POSITIVE	POSITIVE	NEGATIVE	NEGATIVE	NEGATIVE	NEGATIVE	NEGATIVE	POSITIVE	NEGATIVE
622	PICHINCHA	MEDIUM	WARM	POSITIVE	NEGATIVE	NEGATIVE	POSITIVE	NEGATIVE	NEGATIVE	NEGATIVE	NEGATIVE	NEGATIVE	POSITIVE	NEGATIVE
627	PICHINCHA	MEDIUM	WARM	POSITIVE	NEGATIVE	POSITIVE	POSITIVE	NEGATIVE	NEGATIVE	NEGATIVE	NEGATIVE	NEGATIVE	POSITIVE	NEGATIVE
632	PICHINCHA	SMALL	WARM	POSITIVE	NEGATIVE	NEGATIVE	POSITIVE	NEGATIVE	NEGATIVE	NEGATIVE	NEGATIVE	NEGATIVE	NEGATIVE	NEGATIVE
633	PICHINCHA	SMALL	WARM	POSITIVE	NEGATIVE	NEGATIVE	POSITIVE	NEGATIVE	NEGATIVE	NEGATIVE	NEGATIVE	NEGATIVE	POSITIVE	NEGATIVE

Table S2 – Nucleotide Matrix

SAMPLE ID	Pairwise nucleotide identity (%) between Ecuadorian *Acinetobacter* isolates and reference sequences																							
	**NR 026078.1**	**524.2 LAM UDLA ECU**	**522 LAM UDLA ECU**	**522.2 CTR UDLA ECU**	**390.1 CTR UDLA ECU**	**41 CTR UDLA ECU**	**98 CTR UDLA ECU**	**451.1 CTR UDLA ECU**	**99 CTR UDLA ECU**	**93 CTR UDLA ECU**	**97 CTR UDLA ECU**	**598.3 LAM UDLA ECU**	**213.2 LAM UDLA ECU**	**327 LAM UDLA ECU**	**276.2 LAM UDLA ECU**	**506.1 CTR UDLA ECU**	**479.1 CTR UDLA ECU**	**74.3 LS UDLA ECU**	**30.1 CTR UDLA ECU**	**NR 026209.1**	**OR575470.1**	**OL589188.1**	**OR681579.1**	**LC905275.1**
**NR 026078.1**		87,94%	87,91%	88,02%	87,95%	86,45%	86,93%	87,53%	88,02%	86,93%	86,93%	87,40%	86,97%	86,86%	86,93%	88,02%	87,35%	87,94%	87,94%	86,92%	87,80%	87,27%	87,27%	88,02%
**524.2 LAM UDLA ECU**	87,94%		100,00%	99,79%	99,52%	100,00%	88,08%	87,99%	99,79%	88,08%	88,01%	88,18%	88,13%	88,08%	88,01%	99,79%	88,07%	100,00%	100,00%	88,25%	88,25%	88,18%	88,04%	99,79%
**522 LAM UDLA ECU**	87,91%	100,00%		99,79%	99,52%	100,00%	88,08%	87,99%	99,79%	88,08%	88,01%	88,15%	88,13%	88,08%	88,01%	99,79%	88,07%	100,00%	100,00%	88,25%	88,22%	88,15%	88,02%	99,79%
**522.2 CTR UDLA ECU**	88,02%	99,79%	99,79%		99,73%	99,75%	88,16%	88,07%	100,00%	88,16%	88,09%	88,32%	88,21%	88,16%	88,09%	100,00%	88,14%	99,79%	99,79%	88,33%	88,32%	88,26%	88,19%	100,00%
**390.1 CTR UDLA ECU**	87,95%	99,52%	99,52%	99,73%		99,50%	87,95%	87,86%	99,73%	87,95%	87,88%	88,19%	88,00%	87,95%	87,88%	99,73%	88,00%	99,52%	99,52%	88,12%	88,19%	88,05%	88,05%	99,73%
**41 CTR UDLA ECU**	86,45%	100,00%	100,00%	99,75%	99,50%		88,04%	87,76%	99,75%	88,04%	87,95%	88,15%	88,09%	88,04%	87,95%	99,75%	88,02%	100,00%	100,00%	87,91%	88,24%	87,99%	87,99%	99,75%
**98 CTR UDLA ECU**	86,93%	88,08%	88,08%	88,16%	87,95%	88,04%		96,83%	88,16%	99,86%	99,86%	97,40%	100,00%	99,93%	99,86%	88,16%	96,51%	88,08%	88,08%	99,59%	96,51%	96,85%	97,19%	88,16%
**451.1 CTR UDLA ECU**	87,53%	87,99%	87,99%	88,07%	87,86%	87,76%	96,83%		88,07%	96,83%	96,83%	97,41%	97,03%	96,83%	96,83%	88,07%	98,51%	87,99%	87,99%	97,25%	98,50%	100,00%	97,47%	88,07%
**99 CTR UDLA ECU**	88,02%	99,79%	99,79%	100,00%	99,73%	99,75%	88,16%	88,07%		88,16%	88,09%	88,32%	88,21%	88,16%	88,09%	100,00%	88,14%	99,79%	99,79%	88,33%	88,32%	88,26%	88,19%	100,00%
**93 CTR UDLA ECU**	86,93%	88,08%	88,08%	88,16%	87,95%	88,04%	99,86%	96,83%	88,16%		99,93%	97,33%	100,00%	99,86%	99,93%	88,16%	96,58%	88,08%	88,08%	99,52%	96,58%	96,85%	97,26%	88,16%
**97 CTR UDLA ECU**	86,93%	88,01%	88,01%	88,09%	87,88%	87,95%	99,86%	96,83%	88,09%	99,93%		97,40%	100,00%	99,93%	100,00%	88,09%	96,65%	88,01%	88,01%	99,59%	96,64%	96,85%	97,19%	88,09%
**598.3 LAM UDLA ECU**	87,40%	88,18%	88,15%	88,32%	88,19%	88,15%	97,40%	97,41%	88,32%	97,33%	97,40%		97,46%	97,47%	97,40%	88,32%	97,51%	88,18%	88,18%	97,67%	97,65%	97,52%	99,74%	88,32%
**213.2 LAM UDLA ECU**	86,97%	88,13%	88,13%	88,21%	88,00%	88,09%	100,00%	97,03%	88,21%	100,00%	100,00%	97,46%		100,00%	100,00%	88,21%	96,65%	88,13%	88,13%	99,72%	96,64%	97,05%	97,39%	88,21%
**327 LAM UDLA ECU**	86,86%	88,08%	88,08%	88,16%	87,95%	88,04%	99,93%	96,83%	88,16%	99,86%	99,93%	97,47%	100,00%		99,93%	88,16%	96,58%	88,08%	88,08%	99,66%	96,58%	96,85%	97,19%	88,16%
**276.2 LAM UDLA ECU**	86,93%	88,01%	88,01%	88,09%	87,88%	87,95%	99,86%	96,83%	88,09%	99,93%	100,00%	97,40%	100,00%	99,93%		88,09%	96,65%	88,01%	88,01%	99,59%	96,64%	96,85%	97,19%	88,09%
**506.1 CTR UDLA ECU**	88,02%	99,79%	99,79%	100,00%	99,73%	99,75%	88,16%	88,07%	100,00%	88,16%	88,09%	88,32%	88,21%	88,16%	88,09%		88,14%	99,79%	99,79%	88,33%	88,32%	88,26%	88,19%	100,00%
**479.1 CTR UDLA ECU**	87,35%	88,07%	88,07%	88,14%	88,00%	88,02%	96,51%	98,51%	88,14%	96,58%	96,65%	97,51%	96,65%	96,58%	96,65%	88,14%		88,07%	88,07%	96,79%	99,93%	98,51%	97,30%	88,14%
**74.3 LS UDLA ECU**	87,94%	100,00%	100,00%	99,79%	99,52%	100,00%	88,08%	87,99%	99,79%	88,08%	88,01%	88,18%	88,13%	88,08%	88,01%	99,79%	88,07%		100,00%	88,25%	88,25%	88,18%	88,04%	99,79%
**30.1 CTR UDLA ECU**	87,94%	100,00%	100,00%	99,79%	99,52%	100,00%	88,08%	87,99%	99,79%	88,08%	88,01%	88,18%	88,13%	88,08%	88,01%	99,79%	88,07%	100,00%		88,25%	88,25%	88,18%	88,04%	99,79%
**NR 026209.1**	86,92%	88,25%	88,25%	88,33%	88,12%	87,91%	99,59%	97,25%	88,33%	99,52%	99,59%	97,67%	99,72%	99,66%	99,59%	88,33%	96,79%	88,25%	88,25%		96,85%	97,26%	97,53%	88,33%
**OR575470.1**	87,80%	88,25%	88,22%	88,32%	88,19%	88,24%	96,51%	98,50%	88,32%	96,58%	96,64%	97,65%	96,64%	96,58%	96,64%	88,32%	99,93%	88,25%	88,25%	96,85%		98,56%	97,45%	88,32%
**OL589188.1**	88,05%	88,18%	88,15%	88,26%	88,05%	87,99%	96,85%	100,00%	88,26%	96,85%	96,85%	97,52%	97,05%	96,85%	96,85%	88,26%	98,51%	88,18%	88,18%	97,26%	98,56%		97,53%	88,26%
**OR681579.1**	87,27%	88,04%	88,02%	88,19%	88,05%	87,99%	97,19%	97,47%	88,19%	97,26%	97,19%	99,74%	97,39%	97,19%	97,19%	88,19%	97,30%	88,04%	88,04%	97,53%	97,45%	97,53%		88,19%
**LC905275.1**	88,02%	99,79%	99,79%	100,00%	99,73%	99,75%	88,16%	88,07%	100,00%	88,16%	88,09%	88,32%	88,21%	88,16%	88,09%	100,00%	88,14%	99,79%	99,79%	88,33%	88,32%	88,26%	88,19%	

Table S3

Enriched raw milk samples selected for culture-based isolation according to their species-specific PCR.

Target species	Sample	Province	Producer size	Climatic Period	Species-specific PCR targets detected	Culture result	Isolate ID	16S rRNA-based taxonomic assignment
A. baumannii	116	PICHINCHA	MEDIUM	RAINY	*A. baumannii*	Growth compatible with Acinetobacter	522.2 CTR UDLA ECU	A. baumannii
	157	PICHINCHA	SMALL	RAINY	*A. baumannii*	Growth compatible with Acinetobacter	30.1 CTR UDLA ECU	A. baumannii
	175	PICHINCHA	SMALL	RAINY	*A. baumannii*	Growth compatible with Acinetobacter	524.2 LAM UDLA ECU	A. baumannii
	354	PICHINCHA	MEDIUM	WARM	*A. baumannii*	Growth compatible with Acinetobacter	41 CTR UDLA ECU	A. baumannii
	401	MANABÍ	LARGE	WARM	*A. baumannii*	no compatible colonies	N/A	N/A
A. haemolyticus	8	PICHINCHA	SMALL	WARM	*A. haemolyticus; A. lwoffii; A. johnsonii*	no compatible colonies	N/A	N/A
	497	PICHINCHA	MEDIUM	WARM	*A. haemolyticus; A. lwoffii; A. johnsonii*	no compatible colonies	N/A	N/A
	522	MANABÍ	LARGE	WARM	*A. baumannii; A. haemolyticus; A. lwoffii; A. pittii; A. ursingii; A. bereziniae; A. johnsonii*	Growth compatible with Acinetobacter	522 LAM UDLA ECU	A. baumannii
	572	MANABÍ	LARGE	WARM	*A. haemolyticus; A. lwoffii*	Growth compatible with Acinetobacter	598.3 LAM UDLA ECU	A. haemolyticus
	619	PICHINCHA	SMALL	WARM	*A. haemolyticus; A. lwoffii; A. johnsonii*	no compatible colonies	N/A	N/A
A. lwoffii	117	PICHINCHA	SMALL	RAINY	*A. lwoffii*	Growth compatible with Acinetobacter	276.2 LAM UDLA ECU	A. lwoffii
	351	PICHINCHA	LARGE	WARM	*A. lwoffii, A. jhonsonii*	Growth compatible with Acinetobacter	213.2 LAM UDLA ECU	A. lwoffii
	481	MANABÍ	LARGE	WARM	*A. lwoffii*	Growth compatible with Acinetobacter	327 LAM UDLA ECU	A. lwoffii
	557	MANABÍ	MEDIUM	WARM	*A. lwoffii*	no growth	N/A	N/A
	632	PICHINCHA	SMALL	WARM	*A. lwoffii*	no growth	N/A	N/A
A. calcoaceticus	99	MANABÍ	LARGE	RAINY	*A. baumannii; A. lwoffii; A. calcoaceticus; A. pittii; A. johnsonii*	Growth compatible with Acinetobacter	99 CTR UDLA ECU	A. baumannii
	111	PICHINCHA	SMALL	RAINY	*A. lwoffii, A. calcoaceticus, A. jhonsonii*	Growth compatible with Acinetobacter	451.1 CTR UDLA ECU	A. calcoaceticus
	171	PICHINCHA	MEDIUM	RAINY	*A. lwoffii, A. calcoaceticus, A. jhonsonii*	no growth	N/A	N/A
	591	PICHINCHA	SMALL	WARM	*A. lwoffii, A. calcoaceticus, A. jhonsonii*	no compatible colonies	N/A	N/A
	606	PICHINCHA	MEDIUM	WARM	*A. lwoffii, A. calcoaceticus, A. jhonsonii*	no growth	N/A	N/A
A. pittii	93	MANABÍ	SMALL	RAINY	*A. baumannii; A. lwoffii; A. pittii; A. johnsonii*	Growth compatible with Acinetobacter	93 CTR UDLA ECU	A. lwoffii
	106	MANABÍ	SMALL	RAINY	*A. baumannii; A. pittii*	no growth	N/A	N/A
	160	PICHINCHA	MEDIUM	RAINY	*A. baumannii; A. pittii*	Growth compatible with Acinetobacter	479.1 CTR UDLA ECU	A. pitti
	165	PICHINCHA	MEDIUM	RAINY	*A. baumannii; A. pittii*	no growth	N/A	N/A
	168	PICHINCHA	SMALL	RAINY	*A. baumannii; A. pittii*	no compatible colonies	N/A	N/A
A. nosocomialis	18	PICHINCHA	SMALL	WARM	*A. baumannii; A. nosocomialis*	Growth compatible with Acinetobacter	506.1 CTR UDLA ECU	A. lwoffii
	62	PICHINCHA	LARGE	RAINY	*A. baumannii; A. lwoffii; A. nosocomialis; A. jhonsonii*	no growth	N/A	N/A
	74	MANABÍ	SMALL	RAINY	*A. baumannii; A. lwoffii; A. pittii; A. nosocomialis; A. ursingii; A. johnsonii; A. schindleri*	Growth compatible with Acinetobacter	74.3 LS UDLA ECU	A. baumannii
	81	PICHINCHA	SMALL	RAINY	*A. baumannii; A. lwoffii; A. nosocomialis*	no compatible colonies	N/A	N/A
	166	PICHINCHA	MEDIUM	RAINY	*A. baumannii; A. lwoffii; A. nosocomialis*	no growth	N/A	N/A
A. ursingii	4	PICHINCHA	SMALL	WARM	*A. baumannii; A. nosocomialis; A. ursingii; A. johnsonii*	no compatible colonies	N/A	N/A
	209	MANABÍ	SMALL	RAINY	*A. lwoffii; A. ursingii*	no growth	N/A	N/A
	258	MANABÍ	SMALL	WARM	*A. lwoffii; A. ursingii*	no growth	N/A	N/A
	390	PICHINCHA	LARGE	WARM	*A. baumannii; A. lwoffii; A. ursingii; A. johnsonii*	Growth compatible with Acinetobacter	390.1 CTR UDLA ECU	A. baumannii
	479	MANABÍ	SMALL	WARM	*A. lwoffii; A. ursingii; A. pitti*	Growth compatible with Acinetobacter	479.1 CTR UDLA ECU	A. pitti
A. bereziniae	183	PICHINCHA	MEDIUM	RAINY	*A. baumannii; A. lwoffii; A. bereziniae; A. johnsonii*	no compatible colonies	N/A	N/A
	295	MANABÍ	LARGE	WARM	*A. lwoffii; A. ursingii; A. bereziniae; A. johnsonii*	no growth	N/A	N/A
	395	PICHINCHA	MEDIUM	WARM	*A. baumannii; A. lwoffii; A. bereziniae; A. johnsonii*	no compatible colonies	N/A	N/A
	421	MANABÍ	MEDIUM	WARM	*A. baumannii; A. lwoffii; A. ursingii; A. bereziniae; A. johnsonii*	no compatible colonies	N/A	N/A
	567	MANABÍ	MEDIUM	WARM	*A. lwoffii; A. ursingii; A. bereziniae; A. johnsonii*	no growth	N/A	N/A
A. jhonsonii	43	PICHINCHA	SMALL	WARM	*A. baumannii; A. ursingii; A. johnsonii*	no growth	N/A	N/A
	97	MANABÍ	MEDIUM	RAINY	*A. baumannii; A. lwoffii; A. pittii; A. nosocomialis; A. johnsonii*	Growth compatible with Acinetobacter	97 CTR UDLA ECU	A. lwoffii
	212	MANABÍ	SMALL	RAINY	*A. lwoffii, A. jhonsonii*	no growth	N/A	N/A
	355	PICHINCHA	MEDIUM	WARM	*A. baumannii; A. ursingii; A. johnsonii*	no compatible colonies	N/A	N/A
	595	PICHINCHA	MEDIUM	WARM	*A. lwoffii, A. jhonsonii*	no growth	N/A	N/A
A. schindleri	60	PICHINCHA	MEDIUM	RAINY	*A. lwoffii, A. calcoaceticus, A. jhonsonii, A. schindleri, A. pittii, A. baumannii*	Growth compatible with Acinetobacter	98 TR UDLA ECU	A. lwoffii
	178	PICHINCHA	LARGE	RAINY	*A. lwoffii, A. calcoaceticus, A. jhonsonii, A. schindleri, A. pittii, A. baumannii*	no growth	N/A	N/A
	193	PICHINCHA	LARGE	RAINY	*A. lwoffii, A. calcoaceticus, A. jhonsonii, A. schindleri, A. pittii, A. baumannii*	no growth	N/A	N/A
	517	MANABÍ	MEDIUM	WARM	*A. lwoffii, A. calcoaceticus, A. jhonsonii, A. schindleri, A. pittii*	no compatible colonies	N/A	N/A
	553	MANABÍ	MEDIUM	WARM	*A. lwoffii, A. calcoaceticus, A. jhonsonii, A. schindleri*	no compatible colonies	N/A	N/A
